# Metformin induces the AP-1 transcription factor network in normal dermal fibroblasts

**DOI:** 10.1038/s41598-019-41839-1

**Published:** 2019-03-29

**Authors:** Zoe E. Gillespie, Chenxuan Wang, Flaviu Vadan, Topaza Y. Yu, Juan Ausió, Anthony Kusalik, Christopher H. Eskiw

**Affiliations:** 1 Department of Food and Bioproduct Sciences, University of Saskatchewan, Saskatoon, Canada; 2 Department of Biochemistry, University of Saskatchewan, Saskatoon, Canada; 3 Department of Computer Science, University of Saskatchewan, Saskatoon, Canada; 4 Department of Biochemistry and Microbiology, University of Victoria, Victoria, Canada

## Abstract

Metformin is a widely-used treatment for type 2 diabetes and is reported to extend health and lifespan as a caloric restriction (CR) mimetic. Although the benefits of metformin are well documented, the impact of this compound on the function and organization of the genome in normal tissues is unclear. To explore this impact, primary human fibroblasts were treated in culture with metformin resulting in a significant decrease in cell proliferation without evidence of cell death. Furthermore, metformin induced repositioning of chromosomes 10 and 18 within the nuclear volume indicating altered genome organization. Transcriptome analyses from RNA sequencing datasets revealed that alteration in growth profiles and chromosome positioning occurred concomitantly with changes in gene expression profiles. We further identified that different concentrations of metformin induced different transcript profiles; however, significant enrichment in the activator protein 1 (AP-1) transcription factor network was common between the different treatments. Comparative analyses revealed that metformin induced divergent changes in the transcriptome than that of rapamycin, another proposed mimetic of CR. Promoter analysis and chromatin immunoprecipitation assays of genes that changed expression in response to metformin revealed enrichment of the transcriptional regulator forkhead box O3a (FOXO3a) in normal human fibroblasts, but not of the predicted serum response factor (SRF). Therefore, we have demonstrated that metformin has significant impacts on genome organization and function in normal human fibroblasts, different from those of rapamycin, with FOXO3a likely playing a role in this response.

## Introduction

Metformin (MET, N,N- dimethylimidodicarbonimidic diamide) is a well-established and commonly used treatment for type 2 diabetes (T2D)^1^. In recent years, metformin has been documented to have several surprising and beneficial side effects, including use in treating metabolic syndrome^2^, reducing cell proliferation^3–5^, inducing apoptosis in cancer cell lines^6^ and, despite conflicting reports^7^, has been suggested to decrease cancer incidence in T2D patients^8–10^. Metformin has also been reported to have health and lifespan extending benefits in *Caenorhabditis elegans*^11,12^ and *Mus musculus*^13^, although this is controversial as no change in lifespan was observed in *Drosophila melanogaster*^14^ and *Rattus norvegicus*^15^. Despite these beneficial effects, the impact of metformin on the transcriptome of normal healthy cells is poorly understood. Furthermore, the exact mechanisms by which metformin function are poorly characterized.

It is suggested that metformin functions in mammalian systems by inhibiting hepatic gluconeogenesis and increasing muscle sensitivity to insulin^16^. At the cellular level, metformin is proposed to alter the AMP:ATP ratio via mild inhibition of mitochondrial respiratory-chain complex I. This induces phosphorylation of adenosine monophosphate kinase (AMPK)^17,18^, a highly conserved major cellular energy sensor and regulator of homeostasis composed of an α-catalytic and regulatory β and γ subunits. Activation of AMPK indirectly inhibits the mammalian target of rapamycin (mTOR) complex, inducing downstream effects similar to those reported following caloric restriction (CR; reducing nutrient intake without inducing malnutrition) and rapamycin (a commonly used immunosuppressant)^18,19^. Specifically, increased levels of autophagy and decreased protein synthesis, cell growth and proliferation have been reported in response to mTOR inhibition. As such, both rapamycin and metformin have been proposed as mimetics of CR and of one another; however, recent literature has begun to indicate that these compounds may not be direct mimetics of one another. For example, wound healing was accelerated upon application of metformin but not rapamycin in young rodents^20^. Furthermore, rapamycin was shown to decrease neuronal progenitors whereas metformin had no effect whilst still inhibiting mTOR^21^, and both compounds were demonstrated to differentially influence cardiac markers in rats^22^. Despite these findings, the impact of metformin at the transcriptomic level of normal fibroblasts is unknown, as is the extent to which these proposed mimetics differ in influencing the transcriptome.

Genome function, including DNA replication and repair as well as gene expression, is essential for maintaining healthy cells. Changes in gene expression are associated extensively with a wide range of diseases, with expression profiles and pathway analyses used to identify biomarkers and potential therapeutic targets. This is also true for health and longevity: what pathways/mechanisms can we promote to keep our cells healthier for longer? Metformin has numerous reported beneficial effects on health and lifespan, with some previous analyses in *Mus musculus* demonstrating changes in gene expression and a shift towards a CR-like expression profile^13^. These CR-like states function most commonly through inhibition of the mTOR pathway^23^ and although mTOR inhibition and some biochemical impacts of metformin treatment are well characterized, the effects of metformin on genome function (gene expression) and organization (positioning of chromosome territories with the nucleus) in normal human fibroblasts are currently unknown.

Other compounds that are proposed mimetics of CR, such as rapamycin, also promote changes in transcript profiles, as well as repositioning of chromosome territories^24^. Changes in chromosome territory positioning are significant as they represent a large-scale alteration in genome organization. Changes in genome organization have been extensively linked with function. For example, disruption of chromosome territory positioning is a hallmark of the premature aging disease Hutchinson-Gilford Progeria Syndrome (HGPS), with improvement in disease phenotype associated with farnesyl-transferase inhibitor-induced re-location of chromosome territories to resemble those observed in normal fibroblasts^25^. Therefore, understanding genome function and organization in response to metformin (a method of altering nutrient sensing) could provide insight into how altering nutrient sensing can influence three-dimensional folding of the genome.

To determine the impact of altered nutrient sensing via metformin on genome function, primary human foreskin fibroblasts no older than passage 20 (‘young’) were treated with either 0.5 mM or 1.0 mM metformin in culture. Treatments resulted in decreased proliferative rates and decreased numbers of cells actively replicating DNA or exhibiting the proliferative marker Ki67. Metformin treatments also induced re-location of chromosome territories 10 and 18; representing a change in genome organization. RNA sequencing and comparative transcriptome analyses identified changes in genome function in response to metformin treatment, with subsequent network analyses of metformin up-regulated genes revealing enrichment in the AP-1 transcription factor pathway. Comparison of rapamycin to metformin treatment of fibroblasts demonstrated divergence in transcriptome profiles, indicating that these compounds are not direct mimetics of one another. Finally, transcription factor motif analyses identified FOXO3a transcription factor binding sites (TFBS) as over-represented in genes up-regulated by metformin. FOXO3a is a transcription factor that has been identified as involved in the regulation of metabolism, cell proliferation, stress management and life span in addition to its involvement in many diseases^26–29^. Chromatin immunoprecipitation (ChIP) assays further confirm increased FOXO3a binding to specific promoters indicating a functional role of FOXO3a in mediating metformin induced changes in gene expression. These observations indicate differences in the mechanisms governing response to both metformin and rapamycin treatments in normal human fibroblasts.

## Materials and Methods

### Cell Culture, Treatment Conditions and Counts

Normal human foreskin fibroblasts (2DD, described and characterized as normal proliferative dermal fibroblasts in^30,31^), were cultured in 4.5 mg/ml DMEM (Corning, Cat #: ca45000-304), 10% foetal bovine serum (FBS; Gibco, Thermo Fisher Scientific, Cat #: 12483-020) and 1% penicillin/streptomycin (GE Healthcare Life Sciences, Cat #: SV30010). For metformin-treated fibroblasts, media conditions were identical except for the addition of 0.5 mM/1 mM metformin (Santa Cruz Biotechnology, Cat #: sc-202000a) in nuclease free H_2_O. Fibroblasts used were between passage 10 and 20 to ensure that the majority of the cell population was proliferative (2DD populations are known to senesce at passage 50^32^). Furthermore, 2DD cells never exceeded 70% confluence to prevent contact inhibition. For passaging and counting, cells were dissociated from culture flasks using TrypLE Xpress (Life Technologies, Cat #: 12604013) and counted using a haemocytometer. To determine cell viability, cells in suspension were mixed 1:1 with Trypan blue dye (VWR international, Cat #: CA97063-702) and total number of stained and unstained cells recorded. All treatments were conducted for 120 h (selected to use a comparable time point to previous rapamycin-treatments in 2DD), with media changed every 48-72 h to ensure drug presence over time.

### Immunolabelling

To detect the presence of the proliferative marker Ki-67, proliferative, 0.5 mM and 1 mM metformin-treated fibroblasts were grown on coverslips for 120 h before fixing with 4% formaldehyde (FA) in 1X phosphate buffered saline (PBS) for 10 min at RT. Cells were dehydrated in ethanol series (70%, 90%, 100%) and fixed further for 5 min RT in methanol/acetone (1:1 v/v). Cells were rehydrated, washed in 1X PBS and permeabilized with 0.5% Triton X-100/1X PBS for 10 min. Coverslips were blocked in 1% BSA/1X PBS for 20 min before incubation in primary antibody (Rabbit anti-Ki67; Novacastra Cat #: NCL-Ki67p_CE) (1:5,000 dilution in 1% BSA/1X PBS) and secondary antibody for 1 h (goat anti-rabbit A488; Stratech/Jackson Scientific, Cat #: 111–545–003-JIR) (1:200 dilution in 1% BSA/1X PBS). Chromatin was counterstained with Hoechst 33342 (H33342; 1:10,000 dilution in mounting media (Invitrogen, Cat #: h3570)). Coverslips for immunolabelling were fixed in 4% FA/1X PBS, washed in 1X PBS and permeabilized with 0.5% Triton X-100/1X PBS. Coverslips were blocked as above for 20 min in 1X PBS and incubated in primary antibody (Either rabbit anti-LC3 antibody (1:2000 dilution, 1% BSA/1X PBS) Abcam, Cat #: ab48394 or rabbit anti-FOXO3a (1:200 dilution, 1% BSA/1X PBS) Abcam, Cat #: ab12162) and then secondary (goat anti-rabbit 488) for 1 h and mounted as described above.

All images were collected at 40X magnification with a constant exposure time. Gray-scale images were imported into Image J (https://imagej.nih.gov/ij/) and a threshold applied evenly across all images to ensure analyses were unaffected by individual subjectivity. Any nuclei containing label above the threshold were scored as positive for Ki67 or LC3.

### EdU Incorporation

Following 120 h of proliferative growth or 0.5 mM/1.0 mM metformin treatment, live cells were incubated for 2 h with 5 μM 5-ethynyl-2-deoxyuridine (EdU) and fixed as previously described for immunofluorescence. Cells were then labelled using Click-It dye reagents (Life Technologies, Cat #: C10338) following manufacturer’s instructions.

### Flow Cytometry

Proliferative, 0.5 mM and 1 mM metformin-treated fibroblasts were grown for 120 h before fixation in a final concentration of 70% ethanol in 1X PBS. Fixed fibroblasts were pelleted and washed twice in 1X PBS before re-suspension in 1 mL dH_2_O and 20 μL of propidium iodide solution (VWR, Cat #: CA421301-\BL). 100 μg RNase was added and samples incubated at 37 °C for 20 min. BD Accuri C6 flow cytometer was used to analyse samples (Flow rate: slow; 10,000 events). Events were plotted using forward scatter (FSC, X-axis) and side scatter (SSC, Y-axis) parameters to identify single cells. Gates were assigned uniformly across samples to remove cellular debris and doublets from the analysis. A linear histogram plotting fluorescence intensity units (X-axis) against events counted (Y-axis) with gates for each peak were used to identify percentage of cells in G1/G0, S and G2 phase based on DNA content.

### β-Galactosidase Assay

Fibroblasts were grown for 120 h under proliferative, 0.5 mM and 1.0 mM metformin-treated conditions. Cells were grown on glass coverslips in 6-well dishes. Following 120 h, 2DD were stained for β-galactosidase using cell staining protocol and kit from cell signalling (Cell signalling, Cat #: 9860). Briefly, growth media was aspirated and cells were fixed in 1X fixative solution (15 min, RTP) before washing twice in 1X PBS (5 min/wash). 1 ml of β-Galactosidase staining solution was added to each well and incubated overnight at 37 °C in a dry incubator. Cells were imaged by light microscopy. Thresholds for intensity were established and were used to quantify the number of cells present.

### Western Blotting

Western blotting analyses were performed on protein extracts generated from proliferative, 0.5 mM and 1.0 mM metformin-treated fibroblasts at 120 h. Cells were dissociated from culture dishes using TrypLE Express, pelleted and disrupted in RIPA buffer (25 mM Tris-HCl pH 7.6, 150 mM NaCl, 1% NP-40, 1% sodium deoxycholate, 0.1% SDS). 1:100 protease inhibitor cocktail 2 (Sigma-Aldrich, Cat #: P8340) and phosphatase inhibitor cocktail 2 (Sigma-Aldrich, Cat #: P5726) were added to each sample. Equivalent protein extracts were loaded per lane, transferred to nitrocellulose membrane and blocked with 5% skim milk powder in tris-buffered saline containing 0.5% Triton X-100 (SMP/TBST) for 1 h. Primary and secondary antibodies were diluted in 2.5% SMP/TBST. Primary antibodies used were rabbit anti-β-actin (1:1000; Abcam, Cat #: ab8227), rabbit anti-AMPKα1, rabbit anti-AMPKα2, rabbit anti-AMPKthr172 (1:500; Millipore EDM, Cat #:15-115), rabbit anti-LC3 (1:2000; Abcam, Cat #: ab48394), rabbit anti-FOXO3a (1:500; Abcam, Cat #: ab12162) and mouse anti-SRF (1:500; Santa Cruz, Cat #: sc-25290) and were incubated overnight at 4 °C. Secondary antibodies used were either goat anti-rabbit HRP (1:2500; Abcam, Cat #: ab97059) or goat anti-mouse HRP (1:1000; Jackson Scientific, Cat #: 115-035-003) and were incubated for 1 h at RT. Panels of blots in figures were cropped digitally during acquisition. No splicing of information were performed in blots for the same protein.

### 2D Fluorescence *In-Situ* Hybridization/Chromosome Positioning

Proliferative, 0.5 mM and 1.0 mM metformin-treated fibroblasts underwent chromosome painting at 120 h following the protocol detailed by Gillespie *et al*.^24^ and Mehta and colleagues, generating both DOP-PCR of chromosomes to create probes containing biotinylated uridine residues (biotin-16-UTP, Roche) and for the labelling procedure. Nuclei images were captured using a Photometrics cooled charged-coupled device (CCD) camera. A Leica fluorescence microscope (Leitz DMRB) with Plan Fluotar 100 X oil-immersion lens and Smart Capture VP V1.4 were used to collect images. The Cell Nucleus Analyzer (CNA; described previously in Gillespie *et al*.^24^) was used for nuclear segmentation and analysis. At least 50 nuclei were measured for each chromosome under each condition. Briefly, the CNA divides the H33342 stained chromatin into five shells of equal area with the first shell being the outmost and fifth shell being the inner most region of the nucleus. Signal of the probe across shells is normalized by dividing the percentage of probe by the percentage of H33342 signal detected in each shell and mapped to the Y-axis of a bar graph. X-axis represents the shell. Ratios from each shell were averaged and S.E.M. calculated. Students T-tests for unequal variance were conducted to determine significant alterations in chromosome positioning.

### RNA Extractions, cDNA Library Synthesis and RT-qPCR

RNA was extracted from proliferative, 0.5 mM and 1 mM metformin treated fibroblasts at 120 h using RiboZol (Amresco, cat #: N580-100ML) per manufacturer’s instructions. RNA concentration was determined using the NanoDrop2000 (Thermo Scientific) and RNA integrity number (RIN) determined using the Bioanalyzer. RNA with RIN above 8.0 was used for further analysis. To prevent any variation in chronological age or passage number of the culture, fibroblasts for treatment were expanded from a single culture flask. cDNA synthesis for RT-qPCR was conducted using 2 μg of RNA with 50 ng random primers in EasyScript Plus^TM^ RTase reactions following manufacturers instruction (ABM, cat#: G177). The resultant cDNAs were re-suspended in 100 μl dH_2_O. cDNA was further diluted 1:10 in dH_2_O and 1 μl used in each subsequent 10 μl RT-qPCR reaction with 300 mM forward and reverse primer, 1.5 μl H_2_O and 5 μl 2X RT-qPCR mastermix (Life Technologies, Cat #: 4472908). Each reaction was run in triplicate with non-template controls to ensure no contamination. Melt curve analyses were conducted to confirm presence of one product in each reaction. Reactions were conducted using the Rotor Gene RT-qPCR machine (Qiagen). Five normalizing genes (*PRDX5, EFEMP2, FAU, SPARC, FKBP10*) were used in the ΔΔCT method to quantify changes in gene expression. S.E.M were calculated for all genes of interest. A complete list of primers used for RT-qPCR is given (Supplemental Table 1).

### RNA Sequencing and Mapping

Fibroblasts were grown under proliferative, 0.5 mM and 1.0 mM metformin for 120 h. RNA was extracted as previously described and poly-adenylated RNAs purified. cDNA libraries were constructed from 1 μg of RNA using random hexamers to prime the reaction. Average fragment lengths were ~300 bp. Libraries underwent 150 bp paired-end sequencing using the Illumina HiSeq2000 machine. The raw sequencing reads were analysed with FastQC (https://www.bioinformatics.babraham.ac.uk/projects/fastqc/) to establish the initial quality of the RNAseq library. The raw reads were then trimmed with Trim Galore! (https://www.bioinformatics.babraham.ac.uk/projects/trim_galore/) and re-run through FastQC to ensure trimming improved the library quality (example command: trim_galore–phred33 “–outdir = <directory_name>”–paired –illumine –clip_R1 12 –clip_R2 12–length 80 –stringency 2 <forward_read> <reverse_read>). Trimmed reads were mapped to the GRCh38 human reference genome using tophat (example command: tophat–library-type fr-unstranded–coverage-search–microexon-search -p 8 –o <output_directory> <location_of_bowtie2_indices> <forward_trimmed_reads> <reverse_trimmed_reads). Subsequent BAM files were imported into SeqMonk (http://www.bioinformatics.babraham.ac.uk/projects/seqmonk), a tool used for visualization and quantification of RNAseq data. Specifically, 169,714 probes were defined using the Feature Probe Generator. A value of 0.05 was added to the read value of each probe to avoid infinite values during quantification. Examination of several transcripts demonstrated that genes from treated samples had sufficient read depth at ≥2-fold change and these changes were not artefacts of low data volume. Probes were converted to genes in SeqMonk (https://www.bioinformatics.babraham.ac.uk/projects/seqmonk/) and genes that had significant fold changes in treated fibroblasts calculated against proliferative reads as a baseline. Genes were analysed at ≥2-fold cut-off. Two biological replicates were sequenced. RNA-seq files submitted to the Gene Expression Omnibus: GSE104458. Raw rapamycin data sets: GSE65145.

### Network Analysis and CLOVER

Cytoscape with ReactomeFI were utilized in identifying enrichment of network annotation terms (Kyoto Encyclopaedia of Genes and Genomes (KEGG)) to categorize specific pathways enriched in response to either up- or down-regulated genes in either 0.5 mM or 1.0 mM metformin treatments. Lists of Ensembl gene IDs were input to the GeneSet/Mutation Analysis function (2015 network, gene set file format). Built-in functions of ReactomeFI (Analyze Network Functions) were used to identify KEGG and Gene Ontology term enrichment within the constructed networks.

### Chromatin Immuno-precipitation (ChIP)

2DD fibroblasts were grown as described in 150 mm dishes and treated with either 0.5 mM or 1.0 mM metformin for 120 h. The cells were fixed with 1% FA (Electron Microscopy Sciences, Cat #: 15714) in 15 mL of serum-free DMEM media for 10 min at RTP. The reaction was quenched by adding glycine to a final concentration of 125 mM for 10 min at RTP. Supernatant was removed and cells were washed twice in cold 1X PBS and collected by scraping in cold 1X PBS. Cells were pelleted at 200 rcf for 5 min (4 °C), resuspended in 400 μl of lysis buffer (1% SDS, 10 mM EDTA, 50 mM Tris HCl ph 8.0 with 1:100 protease (Sigma-Aldrich, Cat #: P8340) and phosphatase (Sigma-Aldrich, Cat #: P5726) inhibitors) and incubated for 10 min on ice. Samples were sonicated on ice for 1 min at ~30% duty cycle, ~7% output, into chromatin fragments between 200 and 1000 bp in size. Samples were centrifuged at 12,000 rcf, 4 °C for 10 min to pellet debris and supernatant transferred to new 1.5 mL tubes.

For each treatment condition, 30 μl of chromatin was used as input and 60 μl was used for each immunoprecipitation. Chromatin was quantified by nanodrop. 2.5 μg of mouse anti-SRF (Santa Cruz, Cat #: sc-25290) or rabbit anti-FOXO3a (Abcam, Cat #: ab12162) was added to 60 μl of chromatin, diluted 10 times in ChIP buffer (0.01% SDS, 1.1% Triton X100, 1.2 mM EDTA, 16.7 mM Tris-HCl pH 8.0 and 167 mM NaCl) containing Protease Inhibitor Cocktail 2 and Phosphatase Inhibitor Cocktail 2. 2.5 µg donkey anti-mouse HRP was used as the non-specific antibody control. Samples were incubated overnight at 4 °C, rotating before binding of the samples with 50 μl of Dynabeads^TM^ Protein A (Life Technologies, Cat #: 10006D) at 4 °C for 1 h. Samples were subsequently washed for 5 minutes three times with each of: ChIP wash buffer I (0.1% SDS, 1% Triton-X100, 2 mM EDTA, 20 mM Tris pH 8.0 and 150 mM NaCl), ChIP wash buffer II (0.1% SDS, 1% Triton X100, 2 mM EDTA, 20 mM Tris pH 8.0 and 500 mM NaCl) and ChIP wash buffer III (1 mM EDTA and 10 mM Tris-HCl pH 8.0) at RTP. Samples were eluted with 500 μL of freshly made elution buffer (1% SDS and 0.1 M NaHCO3) for 1 h at RTP. For both eluted and inout samples, crosslinks were reversed by adding 200 mM NaCl, 12.5 mM EDTA and 2 μL of proteinase K (Invitrogen, Cat #: 25530049) and incubating with 900 rpm agitation at 65 °C for 5 h. DNA samples were extracted by phenol-chloroform extraction, adding 500 μl phenol chloroform (1:1, pH 8.0), vigorously vortexing and centrifugation (12,000 rcf for 10 min, 4 °C). The upper phase was transferred to a new 1.5 mL tube and DNA precipitated by adding 2.0 μL glycogen, 1X sample volume isopropanol. Samples were then centrifuged (12,000 rcf, 30 min, 4 °C) and supernatant discarded. The resultant DNA pellet from the input sample was re-suspended in 40 µL nuclease-free water and DNA from immuno-precipitated samples was re-suspended in 80 µL nuclease-free water. Chromatin shearing efficiency was monitored by running 5 µL input DNA samples on a 1.5% agarose gel. qPCR was performed in 10 μL reactions containing 5 μL PerfeCTa® SYBR® Green SuperMix for iQ (Quantabio, Cat #: 95053-500), 1 μL ChIP DNA sample, 3 μL H2O and 1 μL of 3 μM forward and reverse ChIP primers. All reactions for each gene were run in triplicate with non-template controls. ChIP-qPCR data was normalized by the percent input method. ChIP-qPCR primers used are listed in Supplemental Table 2).

## Results

### Metformin reduces proliferative rates in normal human fibroblasts

**Figure 1:**
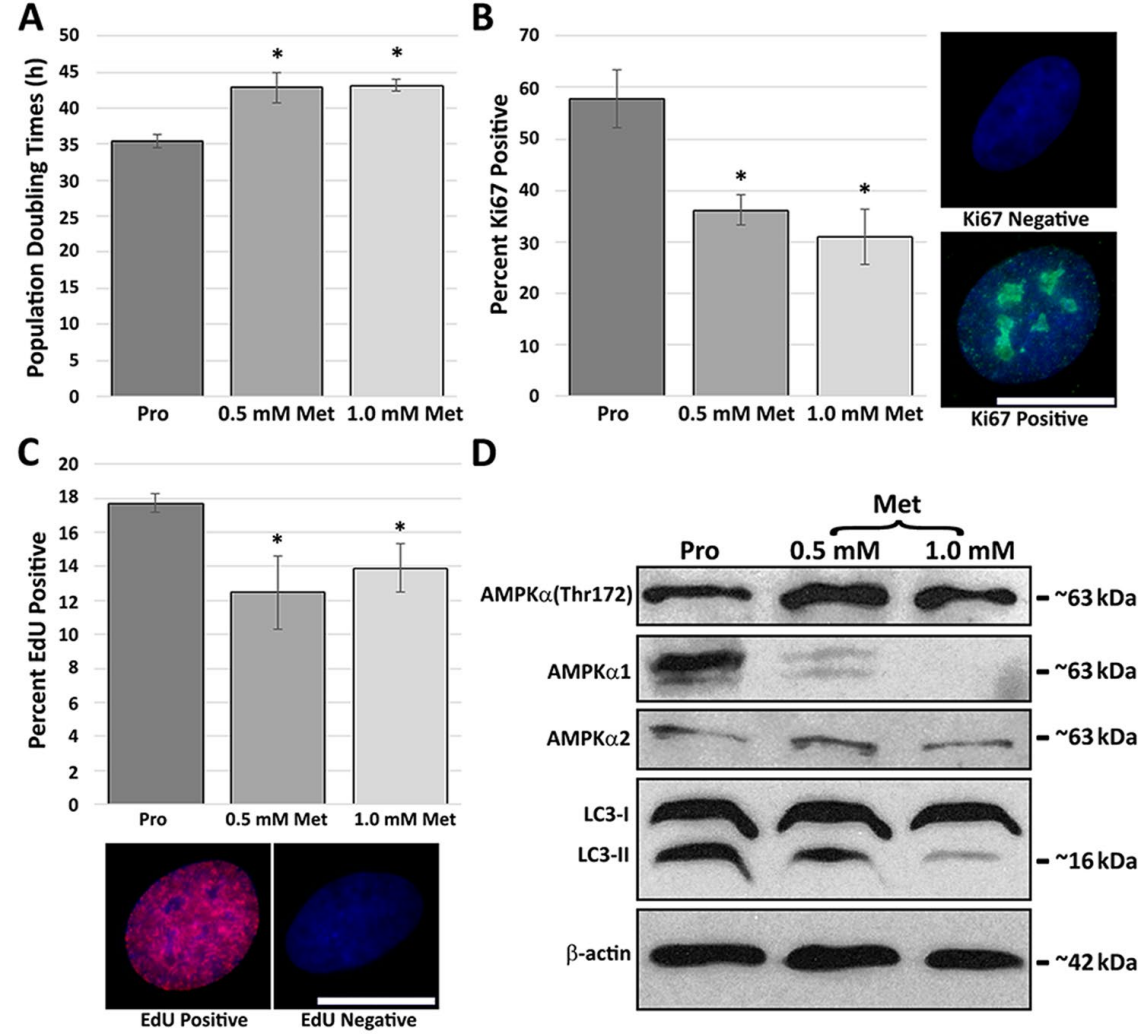
Metformin decreases the rate of fibroblast proliferation. (**A**) 2DD fibroblasts were grown under normal culture conditions or in the presence of 0.5/1.0 mM metformin. Cell numbers were monitored and population doubling times (Y axis) calculated at 120 h. (**B**) The total population doublings (Y axis) for 0.5 mM and 1.0 mM metformin treatments (X axis) were calculated. 2DD were immuno-labelled for Ki67 following 120 h of treatment. (**C**) Percent Ki67 positive (Y axis) for 0.5 mM/1.0 mM metformin (X-axis) is plotted. Ki67 positive and negative cells are shown at the bottom of the panel. Ki67 is false coloured green and chromatin counterstained with H33342 (blue). (**D**) Percent EdU positive (Y axis) 2DD fibroblasts at 120 h following 0.5 mM/1.0 mM metformin treatment. Below the panel, EdU positive and negative fibroblasts are shown. EdU is false coloured red and chromatin counterstained with H33342 (blue). Data represent three biological replicates. Error bars represent S.E.M. Scale bars = 10 μm.

To determine the impact of metformin on proliferating cells, proliferating primary human dermal foreskin fibroblasts (designated 2DD) were treated with either 0.5 mM or 1.0 mM metformin for 120 h. These concentrations were selected as they showed effects on cell growth in culture. 120 h was selected to provide comparability to previously published 120 h 500 nM rapamycin treatments. Higher concentrations of metformin have been frequently used^33^; however, we aimed to keep our concentrations close to physiologically relevant^34^ while still showing an impact on growth profiles without cell death. At 120 h, population doubling times within culture were calculated (Fig. 1A). We observed a significant increase (p < 0.05) in the doubling times from an average of 35.5 h under proliferating conditions to 42.9 h and 43.2 h under 0.5 mM and 1.0 mM respectively following 120 h metformin treatment. Furthermore, cultures were confirmed to be mostly proliferative by β-galactosidase assays (proliferative 3.48% β-gal positive, 0.5 mM metformin 3.02% β-gal positive and 1.0 mM metformin-treated 3.85% β-galactosidase positive) and neither concentration of metformin induced an increase in cell death as determined by Trypan Blue, with proliferative samples exhibiting 95.9% cell survival, 0.5 mM metformin-treated samples had 96.6% survival, and 1.0 mM metformin treatment had 98.6% survival (Supplemental Fig. 3).

To further determine the impact of metformin treatment on cell proliferation the percentage of 2DD cells exhibiting Ki67, a protein marker of actively proliferating cells, was determined (Fig. 1C). In response to 0.5 mM and 1.0 mM metformin treatment, the percentage of cells exhibiting Ki67 decreased significantly from 57.8% to 36.1% and 31.02% respectively. In agreement with this, the number of cells actively progressing through S-phase, as determined by incorporation of the thymine analogue EdU, decreased from 17.7% in proliferative samples to 12.5% in 0.5 mM metformin-treated fibroblasts and 13.9% in 1.0 mM metformin-treated fibroblasts (Fig. 1B,C). Cell cycle analysis by flow cytometry confirmed these findings, with cells in G1/G0 increasing in response to 0.5 mM and 1.0 mM metformin treatment from 74.0% to 79.0% and 78.8% respectively, and the number of cells in S-phase decreased from 13.3% in proliferative samples to 11.8% and 11.9% in 0.5 mM and 1.0 mM metformin treated samples (Supplemental Fig. 3). Western blot analyses were used to confirm that our metformin treatments were functioning through the expected biochemical pathways, increasing phosphorylated AMPKα(Thr172) and decreasing the marker of autophagy, LC3-II (Fig. 1D). These findings indicate that metformin is causing a reduction in growth rates within cells without causing cell death by interfering with AMP/ATP ratios.

### Metformin induced changes in genome organization (chromosome territory positioning)

**Figure 2:**
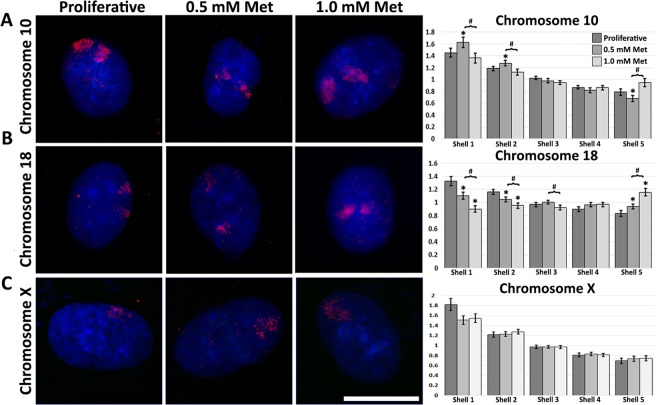
Chromosome territories re-locate following 0.5 mM/1.0 mM metformin treatment. Chromosomes (**A**) 10, (**B**) 18, (**C**) X were identified in proliferative (Proliferative, first column), 0.5 mM metformin-treated (0.5 mM Met, second column) and 1.0 mM metformin-treated (1.0 mM Met, third column) 2DD fibroblasts by chromosome painting. Red signal represents the identified chromosomes; chromatin was counter stained with H33342 (blue). Scale bar = 10 μm. Cell Nucleus Analyser (CNA) software broke nuclei into five concentric shells of area, shell 1 being the most exterior and 5 the most interior (X-axis). Y-axes of graphs for each chromosome (X, 10, 18) represent the measured ratio of % chromosome signal/% H333432 signal in each shell. This ratio normalizes for DNA content in each shell. Error bars = S.E.M. *p-value ≤ 0.05 between treatment and proliferative. ^#^Significant difference (p-value ≤ 0.05) between 0.5 mM and 1.0 mM metformin.

Previous work from ourselves and others have demonstrated that changes in growth factor availability or disruption of cellular energy/nutrient sensing causes the re-organization of chromosomes concomitantly with changes in gene expression^24,31,35^. To determine if 0.5 mM/1.0 mM metformin treatments were having an impact on genome organization at 120 h, we labelled specific chromosomes using 2D-DNA fluorescence *in-situ* hybridization (FISH) to identify and analyse re-localization of chromosomes 10 and 18. This technique allows the “painting” of specific chromosomes, which can then be used to determine nuclear location. Following imaging, erosion analysis, which divides the nucleus into 5 concentric rings of equal area, with the outer most being the nuclear periphery (shell 1) and the inner most the nuclear interior (shell 5), was used to determine the location of these chromosomes within the nuclear volume. The percent of each “painted” chromosome that fell into each shell was measured and normalized by dividing by the counter stained signal for chromatin in each shell. In 0.5 mM and 1.0 mM metformin-treated fibroblasts, we observed significant changes in genome organization with re-location of chromosomes 10 and 18 within the nuclear volume (Fig. 2A,B). 0.5 mM metformin induced statistically significant (Student t-test P < 0.05) re-positioning of chromosomes 10 and 18, 10 towards the nuclear periphery and 18 towards the nuclear interior, a similar pattern, but less dramatic response to, that observed following 500 nM rapamycin treatment. Interestingly, in 1.0 mM metformin-treated samples, repositioning of chromosome 10 was not significant; however, like with 0.5 mM, chromosome 18 also re-positioned to the nuclear interior. Chromosome X was used as a control and no significant change in positioning was documented, remaining at the nuclear periphery (Fig. 2C). Furthermore, comparisons between 0.5 mM and 1.0 mM metformin treatments also revealed significant differences in nuclear locations of chromosomes 10 and 18 (P-value < 0.05). Therefore, we identified significant changes in chromosome territory positioning of chromosomes 10 and 18 in response to metformin when 0.5 mM metformin and 1.0 mM metformin treatments were compared to proliferative samples, and when metformin treatments were compared with one another. These observations indicate that metformin caused primary fibroblasts to re-organize their genome through repositioning of chromosomes within the nuclear volume.

### Metformin induces concentration dependent changes in transcript profiles

**Figure 3:**
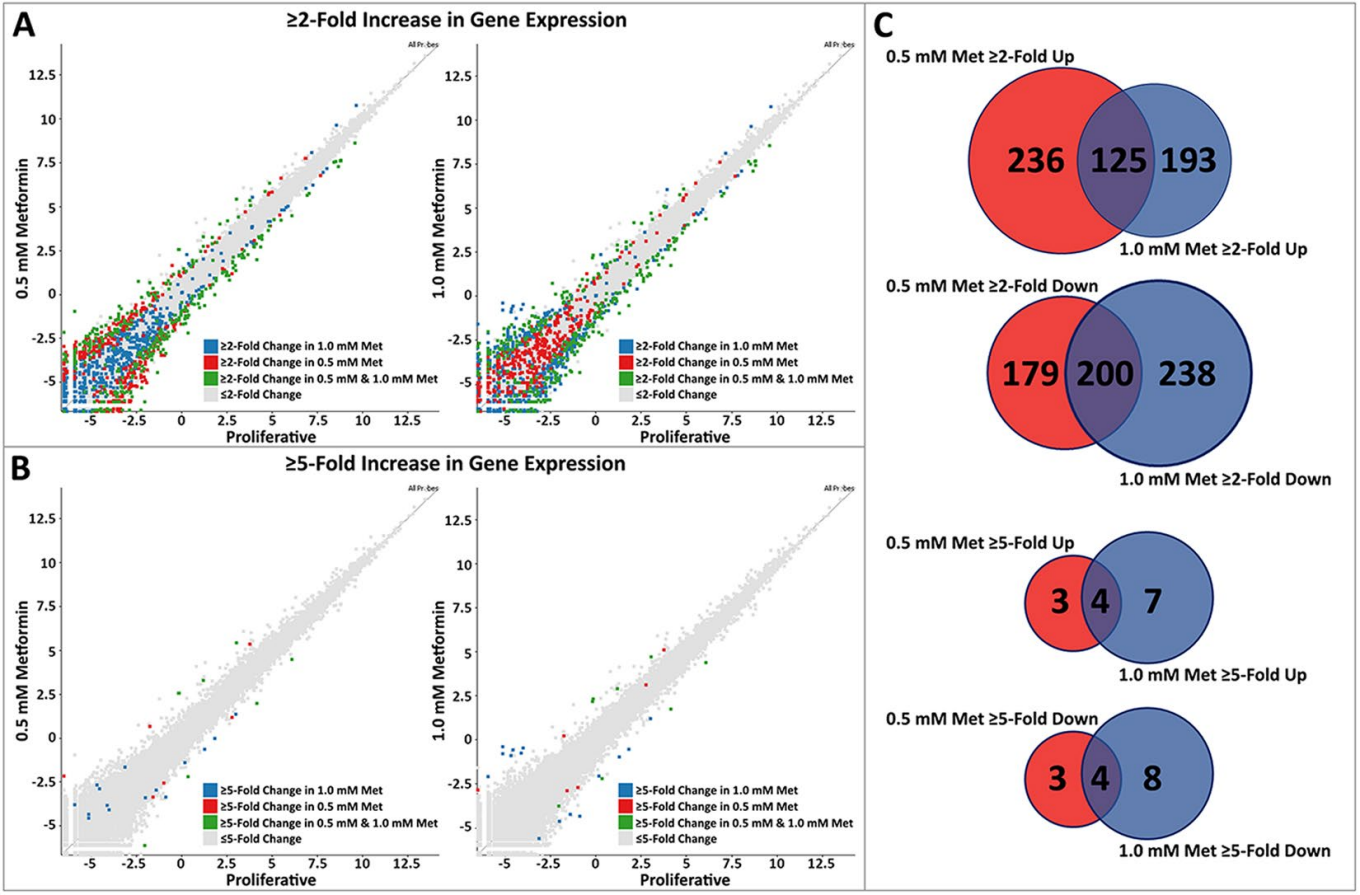
Scatter plots demonstrating transcriptome profile in 0.5 mM/1.0 mM metformin treatments. (**A**) Scatter plot comparing the transcript abundance in 0.5 mM metformin (Y-axis) and proliferating (X-axis) fibroblasts. (**B**) Scatter plot comparing the transcript abundance in 1.0 mM metformin (Y-axis) and proliferating (X-axis) fibroblasts. Counts identified for each transcript by RNA-seq for proliferative were log-base-2 transformed. Each square represents a single transcript. Transcripts exhibiting ≥2-fold change in 0.5 mM metformin-treated fibroblasts when compared to proliferative are marked in blue. Transcripts exhibiting ≥2-fold change in 1.0 mM metformin-treated fibroblasts when compared to proliferative are marked in red. Gray squares represent transcripts that did not change abundance ≥2-fold. Green squares represent transcripts that had a ≥2-fold change in response to both 0.5 mM and 1.0 mM metformin when compared to proliferative fibroblasts. Black text highlights individual transcripts within each scatter plot. (**C**) Venn diagrams demonstrating the number of genes up-regulated (left) or down-regulated (right) shared between 0.5 mM (red) and 1.0 mM (blue) metformin treatments. Numbers in each segment represent genes that are not shared, and in overlapping segments, shared genes between 0.5 mM and 1.0 mM metformin-treated fibroblasts.

As we had observed that metformin induced changes in proliferative rates as well as resulting in re-positioning of chromosomes 10 and 18 within the nuclear volume, our next aim was to determine if this equated to changes in transcript profiles. We performed comparative transcriptome analyses of RNA sequencing data generated from mRNAs isolated from 0.5 mM and 1.0 mM metformin treated 2DD fibroblasts. Raw sequence reads from Illumina-based sequencing were mapped to a reference genome (GRCh38/hg38 assembly from Ensembl), normalized using RPM (reads per million) and analysed. For initial analyses, scatter plots were generated, indicating the log number of reads from 0.5 mM metformin-treated fibroblasts against the log number of reads from proliferative fibroblasts. A subset of genes changing ≥2-fold were highlighted in 0.5 mM metformin-treated vs proliferative fibroblasts and in 1.0 mM metformin-treated vs proliferative fibroblasts (Fig. 3A). Additional analyses were also conducted for genes changing expression greater than ≥5-fold (Fig. 3B). In total, 740 genes changed expression ≥2-fold in response to 0.5 mM metformin treatments (361 up-regulated; 379 down-regulated) whilst 754 genes changed expression ≥2 fold in response to 1.0 mM metformin treatment (318 up-regulated; 438 down-regulated). When applying a more stringent ≥5-fold cut-off, 14 genes changed expression in response to 0.5 mM metformin (7 up-regulated; 7 down-regulated) and 23 genes changed expression in response to 1.0 mM metformin (11 up-regulated; 12 down-regulated) (Supplemental Table 4). Although genes from both 0.5 mM and 1.0 mM metformin treatments had similar read count values to those of proliferative cells (correlations of 0.996 for both), scatter plots indicated there was some divergence between genes changing expression between 0.5 mM and 1.0 mM metformin treatments. Examination of 0.5 mM and 1.0 mM metformin gene lists at ≥2-fold up-regulated changes in gene expression revealed 125/552 up-regulated genes (22%) of genes changing expression overlapping between the two treatments, and 200/617 genes (32%) of genes overlapping between 0.5 mM and 1.0 mM metformin down-regulated genes (Fig. 3C). At ≥5-fold up, 4/14 (28% genes) continued to overlap, and ≥5-fold down, 4/15 (27%) of genes overlapped between 0.5 mM and 1.0 mM metformin treated samples (Fig. 3C).

Changes in gene expression from both 0.5 mM and 1.0 mM metformin treated fibroblasts were validated by reverse transcriptase quantitative PCR (RT-qPCR) to ensure observations from RNA-seq were accurate. Of the 25 genes analysed to have changed expression by RNA-seq in response to 0.5 mM or 1.0 mM metformin, 19 and 17 (respectively) also changed by qRT-PCR (Supplemental Fig. 5). Negative controls were selected from the RNAseq data as not changing expression and remained unchanged when measured by RT-qPCR. These observations indicate that there are quantifiable changes in gene expression in primary fibroblasts following metformin treatment and that these changes are dependent on the concentration used.

### Network analyses of metformin-treated fibroblasts revealed enrichment in the AP-1 transcription factor and cytokine-cytokine interaction pathways

**Figure 4:**
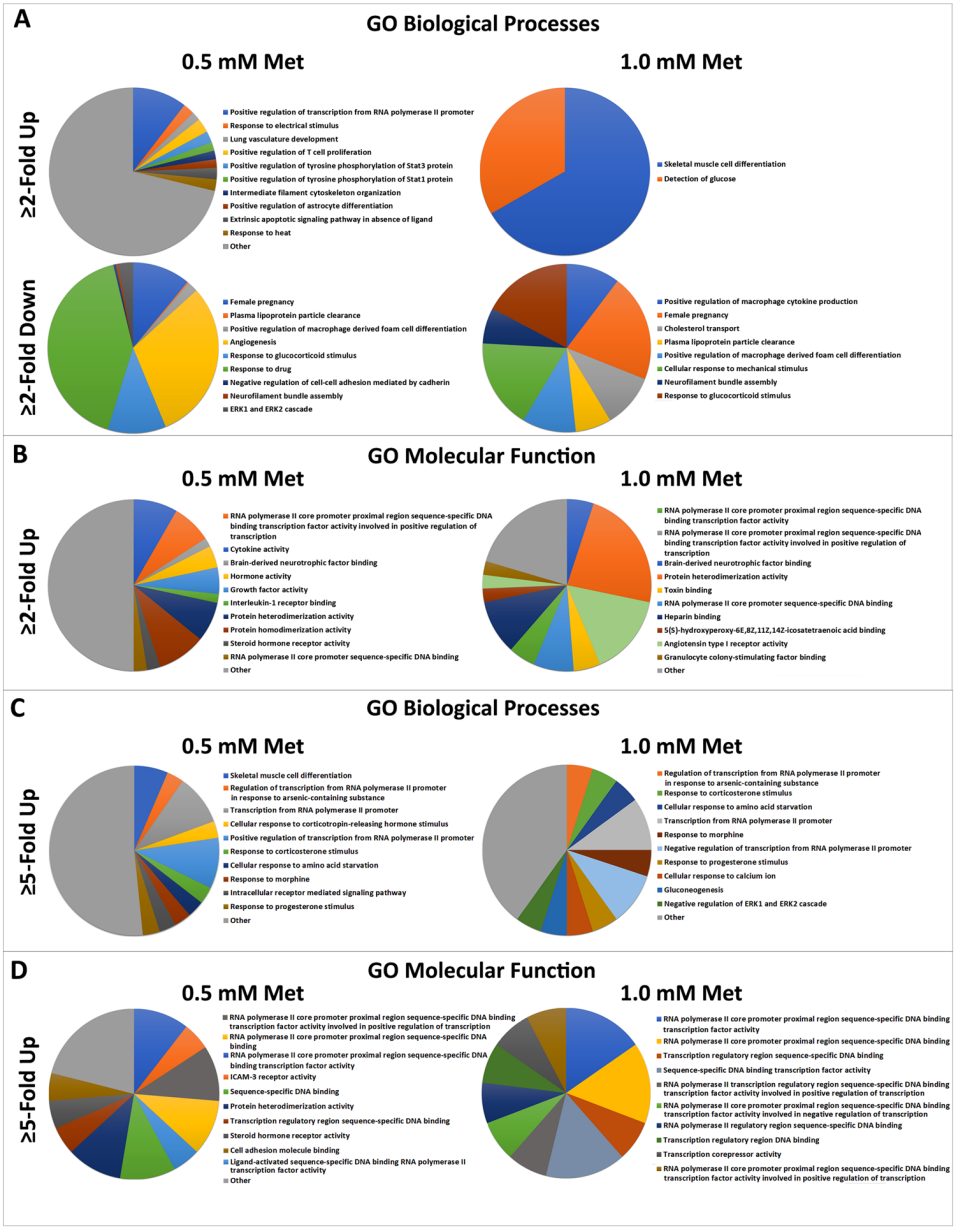
Gene Ontology (GO) terms for genes changing ≥2-fold and ≥5-fold in response to 0.5 mM and 1.0 mM metformin treatments in 2DD fibroblasts. (**A**) Pie charts visualizing GO Biological Processes in response to genes up and down-regulated ≥2-fold in response to 0.5 mM (left) and 1.0 mM (right) metformin. (**B**) Pie charts visualizing GO Molecular Function in response to genes up-regulated ≥2-fold in response to 0.5 mM (left) and 1.0 mM (right) metformin. (**C**) Pie charts visualizing GO Biological Processes in response to genes up regulated ≥5-fold in response to 0.5 mM (left) and 1.0 mM (right) metformin. (**D**) Pie charts visualizing GO Molecular Function in response to genes up-regulated ≥5-fold in response to 0.5 mM (left) and 1.0 mM (right) metformin. All data presented had an FDR and P-value < 0.05. Data for go terms of genes down-regulated ≥5-fold in response to 0.5 mM or 1.0 mM metformin are not presented as no terms met our threshold for significance. Keys corresponding colours to GO terms on the pie charts are given to the right of the chart.

To determine the biological pathways enriched in response to metformin treatments, network analyses were conducted on gene sets identified to change expression in response to either 0.5 mM or 1.0 mM metformin for 120 h. Cytoscape with ReactomeFI was used to classify interacting groups of genes (modules) from our data sets and to identify the associated pathway annotation terms. In response to 0.5 mM metformin-treatment, 6 unique clusters of associated genes, or modules, were identified in genes ≥2-fold up-regulated genes (Supplemental Fig. 6). Gene set enrichment analysis (GSEA) and subsequent pathway analyses of these modules revealed significant enrichment in the AP-1 transcription factor pathway, alongside several of its associated KEGG pathways, including but not limited to; the ATF-2 (activating transcription factor 2) networks, validated transcriptional targets of AP1 family members Fra1 and Fra2 (FOS related antigen 1/2) and IL6-mediated signalling events. The TNF signalling pathway and cytokine-cytokine receptor interaction pathways were also among of the top results for enrichment in response to 0.5 mM metformin treatment (Table 1). Analysis of 0.5 mM metformin ≥2-fold up-regulated genes demonstrated enrichment for Gene Ontology (GO) terms for molecular processes and biological function including: positive regulation of transcription from RNA polymerase II promoters, sequestering of triglyceride and cytokine activity (Fig. 4A,B).

**Table 1:** Biological pathway enrichment of networks constructed from genes ≥2 fold upregulated in response to 0.5 mM metformin.

Gene Set	Protein from Network	P-Value	FDR	Nodes
AP-1 transcription factor network(N)	3	3.19E-06	4.11E-04	FOSB, FOS, ATF3
HTLV-I infection(K)	3	1.55E-04	9.93E-03	FOS, ITGAL0, ATF3
ATF-2 transcription factor network(N)	2	3.19E-04	0.0136	FOS, ATF3
Amphetamine addiction(K)	2	4.25E-04	0.0136	FOSB, FOS
Glucocorticoid receptor regulatory network(N)	2	5.74E-04	0.0144	NR4A1, FOS
Rheumatoid arthritis(K)	2	7.46E-04	0.0157	FOS, ITGAL
Osteoclast differentiation(K)	2	1.60E-03	0.0289	FOSB, FOS
Fosb gene expression and drug abuse(B)	1	2.45E-03	0.03	FOSB
Tsp-1 induced apoptosis in microvascular endothelial cell(B)	1	3.91E-03	0.03	FOS
Pertussis toxin-insensitive ccr5 signaling in macrophage(B)	1	4.40E-03	0.03	FOS
MAPK signaling pathway(K)	2	5.93E-03	0.03	NR4A1, FOS
Repression of pain sensation by the transcriptional regulator dream(B)	1	6.35E-03	0.03	FOS
Il 3 signaling pathway(B)	1	6.35E-03	0.03	FOS
Ras signaling in the CD4+ TCR pathway(N)	1	6.84E-03	0.03	FOS
Calcium signaling by hbx of hepatitis b virus(B)	1	6.84E-03	0.03	FOS
Oxidative stress induced gene expression via nrf2(B)	1	6.84E-03	0.03	FOS
Cadmium induces dna synthesis and proliferation in macrophages(B)	1	7.32E-03	0.03	FOS
Bone remodeling(B)	1	7.81E-03	0.03	FOS
Nerve growth factor pathway (ngf)(B)	1	8.30E-03	0.03	FOS
Mets affect on macrophage differentiation(B)	1	8.78E-03	0.03	FOS
PDGFR-alpha signaling pathway(N)	1	0.0102	0.03	FOS
Igf-1 signaling pathway(B)	1	0.0107	0.03	FOS
Inhibition of cellular proliferation by gleevec(B)	1	0.0117	0.03	FOS
Role of mef2d in t-cell apoptosis(B)	1	0.0122	0.03	NR4A1
Tpo signaling pathway(B)	1	0.0122	0.03	FOS

The enriched pathway annotation terms (GeneSet) are listed. P-value and false discovery rates are presented. Nodes lists genes from our data set present in the enriched pathway.

Six unique modules were identified in genes ≥2-fold down-regulated in response to 0.5 mM metformin (Supplemental Fig. 6), with pathways analyses demonstrated no enriched pathways. Down-regulated GO biological processes included: positive regulation of inflammatory response and positive regulation of reactive oxygen species and metabolic processes. These observations indicate that metformin is likely driving the up-regulation of biological functions through changes in gene expression.

In genes ≥2-fold up-regulated in response to 1.0 mM metformin treatments, 6 distinct modules were identified, no significant pathway enrichment was observed (Supplemental Fig. 6). Although, pathway analyses of these modules still included all enriched pathways identified from genes ≥2-fold up-regulated in 0.5 mM metformin treatments, with the exception of the Interleukin signalling pathway. However, with the same analytical parameters at genes ≥5-fold up-regulated by either 0.5 mM or 1.0 mM metformin treatments, the AP-1 transcription factor pathway was enriched (Tables 2, 3). Analysis of GO terms for molecular processes and biological function also included positive regulation positive regulation of transcription from RNA polymerase II promoters, with GO term detection of glucose unique to 1.0 mM metformin treatments (Fig. 4B). Identical analyses with 1.0 mM metformin genes ≥5-fold up-regulated genes revealed additional GO terms, including cellular response to amino acid starvation and gluconeogenesis (Fig. 4C,D). (GeneSet) are listed. P-value and false discovery rates are presented. Nodes lists genes from our data set present in the enriched pathway.

**Table 2:** Biological pathway enrichment of networks constructed from genes ≥5-fold up-regulated in response to 0.5 mM metformin.

Gene Set	Protein From Network	P-Value	FDR	Nodes
AP-1 transcription factor network(N)	7	2.57E-06	1.04E-03	JUNB, ATF3, EGR1, DUSP1, FOS, FOSB, IL6
ATF-2 transcription factor network(N)	6	1.14E-05	2.32E-03	JUNB, ATF3, DUSP1, FOS, IL6, HES1
Downstream signaling in naïve CD8+ T cells(N)	6	1.98E-05	2.67E-03	JUNB, EGR1, FOS, TNF, IFNA10, TNFRSF18
TNF signaling pathway(K)	7	4.63E-05	4.08E-03	JUNB, LIF, FOS, IL1B, TNF, VCAM1, IL6
Calcineurin-regulated NFAT-dependent transcription in lymphocytes(N)	5	5.04E-05	4.08E-03	JUNB, EGR1, EGR3, FOS, TNF
Malaria(K)	5	6.77E-05	4.54E-03	IL1B, TNF, VCAM1, IL6, ITGAL
Cytokine-cytokine receptor interaction(K)	10	8.94E-05	5.19E-03	LIF, IL21, IL1A, IL1B, TNF, IFNA10, THPO, IL6, TNFRSF18, CCL8
Rheumatoid arthritis(K)	6	1.21E-04	6.05E-03	FOS, IL1A, IL1B, TNF, IL6, ITGAL
African trypanosomiasis(K)	4	2.19E-04	8.26E-03	IL1B, TNF, VCAM1, IL6
Prion diseases(K)	4	2.45E-04	8.26E-03	EGR1, IL1A, IL1B, IL6
signal transduction through il1r(B)	4	2.45E-04	8.26E-03	FOS, IL1A, IL1B, TNF
Inflammatory bowel disease (IBD)(K)	5	2.50E-04	8.26E-03	IL21, IL1A, IL1B, TNF, IL6
HTLV-I infection(K)	9	3.64E-04	0.0113	ATF3, EGR1, FOS, TNF, ZFP36, CCND2, VCAM1, IL6, ITGAL
Graft-versus-host disease(K)	4	4.43E-04	0.0129	IL1A, IL1B, TNF, IL6
Pertussis(K)	5	4.79E-04	0.0129	FOS, IL1A, IL1B, TNF, IL6
Cellular roles of Anthrax toxin(N)	3	6.07E-04	0.0152	IL1B, TNF, VCAM1
Osteoclast differentiation(K)	6	9.27E-04	0.0204	JUNB, FOS, IL1A, IL1B, FOSB, TNF
Hematopoietic cell lineage(K)	5	9.29E-04	0.0204	IL1A, IL1B, TNF, THPO, IL6
IL27-mediated signaling events(N)	3	1.49E-03	0.0306	IL1B, TNF, IL6
MAPK signaling pathway(K)	8	1.53E-03	0.0306	DUSP1, FOS, IL1A, IL1B, TNF, FGF9, NR4A1, NTRK2
Chagas disease (American trypanosomiasis)(K)	5	2.03E-03	0.0385	FOS, IL1B, TNF, GNAO1, IL6
Toll-like receptor signaling pathway(K)	5	2.20E-03	0.0396	FOS, IL1B, TNF, IFNA10, IL6
Jak-STAT signaling pathway(K)	6	2.38E-03	0.0404	LIF, IL21, CCND2, IFNA10, THPO, IL6

The enriched pathway annotation terms (GeneSet) are listed. P-value and false discovery rates are presented. Nodes lists genes from our data set present in the enriched pathway.

**Table 3:** Biological pathway enrichment of networks constructed from genes ≥5 fold up-regulated in response to 1.0 mM metformin.

Gene Set	Protein from Network	P-Value	FDR	Nodes
AP-1 transcription factor network(N)	3	3.22E-07	3.06E-05	FOSB, FOS, ATF3
ATF-2 transcription factor network(N)	2	9.64E-05	3.98E-03	FOS, ATF3
Amphetamine addiction(K)	2	1.29E-04	3.98E-03	FOSB, FOS
Osteoclast differentiation(K)	2	4.89E-04	0.0113	FOSB, FOS
Fosb gene expression and drug abuse(B)	1	1.47E-03	0.0152	FOSB
HTLV-I infection(K)	2	1.88E-03	0.0152	FOS, ATF3
Tsp-1 induced apoptosis in microvascular endothelial cell(B)	1	2.35E-03	0.0152	FOS
Pertussis toxin-insensitive ccr5 signaling in macrophage(B)	1	2.64E-03	0.0152	FOS
Repression of pain sensation by the transcriptional regulator dream(B)	1	3.81E-03	0.0152	FOS
Il 3 signaling pathway(B)	1	3.81E-03	0.0152	FOS
Calcium signaling by hbx of hepatitis b virus(B)	1	4.11E-03	0.0152	FOS
Ras signaling in the CD4+ TCR pathway(N)	1	4.11E-03	0.0152	FOS
Oxidative stress induced gene expression via nrf2(B)	1	4.11E-03	0.0152	FOS
Cadmium induces dna synthesis and proliferation in macrophages(B)	1	4.40E-03	0.0152	FOS
Bone remodeling(B)	1	4.69E-03	0.0152	FOS
Nerve growth factor pathway (ngf)(B)	1	4.99E-03	0.0152	FOS
Mets affect on macrophage differentiation(B)	1	5.28E-03	0.0152	FOS
PDGFR-alpha signaling pathway(N)	1	6.16E-03	0.0152	FOS
Igf-1 signaling pathway(B)	1	6.45E-03	0.0152	FOS
Inhibition of cellular proliferation by gleevec(B)	1	7.03E-03	0.0152	FOS
Tpo signaling pathway(B)	1	7.33E-03	0.0152	FOS
Nongenotropic Androgen signaling(N)	1	7.33E-03	0.0152	FOS
S1P2 pathway(N)	1	7.62E-03	0.0152	FOS
Calcium signaling in the CD4+ TCR pathway(N)	1	7.91E-03	0.0152	FOS
Bcr signaling pathway(B)	1	7.91E-03	0.0152	FOS

The enriched pathway annotation terms (GeneSet) are listed. P-value and false discovery rates are presented. Nodes lists genes from our data set present in the enriched pathway.

Seven unique modules were identified in genes ≥2-fold down-regulated in response to 1.0 mM metformin (Supplemental Fig. 6); however, in agreement with pathway analyses from 0.5 mM metformin genes ≥2-fold down-regulated, no pathway enrichment was detected. GO enrichment revealed 4 GO biological terms shared by 0.5 mM and 1.0 mM metformin down-regulated genes, with no down-regulation of positive regulation of inflammatory response and positive regulation of reactive oxygen species and metabolic processes, although the GO term for inflammatory response was enriched. Despite enrichment of cytokines in gene lists and cytokine-associated pathways in pathway analyses, removal of metformin treatment resulted in cells increasing their proliferative rates (data not shown). This indicates that the increase in cytokine expression associated with metformin is not inducing senescence and that that cytokine gene transcription is not related to the senescence associated secretory phenotype (SASP) pathway.

Pathway analyses of overlapping genes between 0.5 mM and 1.0 mm metformin further support the AP-1 transcription factor pathway as a key response to metformin-treatments. Whilst no pathways were enriched in genes overlapping between down-regulated gene-sets (Supplemental 7, 9), analysis of genes up-regulated ≥2-fold and common to both 0.5 mM and 1.0 mM metformin treatments revealed enrichment of the AP-1 transcription factor pathway (FDR ≤ 0.05; p-value ≤ 0.05) (Supplemental Tables 8, 9). Up-regulated genes not shared between metformin concentrations had no common pathway enrichment whilst down-regulated unshared genes had no pathway enrichment (FDR ≤ 0.05; p-value ≤ 0.05). This indicates that a core set of genes are important for the response of fibroblasts to metformin.

### Metformin and rapamycin induce different gene expression profiles

As metformin and rapamycin are proposed mimetics of caloric restriction, it has been suggested that they mimic one another. To determine the overlap in the biological impact of metformin and rapamycin on genome function, we conducted meta-analyses of data collected from 2DD fibroblasts treated for 120 h with 500 nM rapamycin and compared these analyses to our current 0.5 mM/1.0 mM 120 h metformin-treated 2DD fibroblasts^24^. Previously, we reported that rapamycin treatment increases population doubling times and decreases cell proliferation^24^. Similarly, 0.5 mM/1.0 mM metformin treatments induced significant increases in population doubling times and decreases in cell proliferation; however, changes were far more pronounced in rapamycin-treated fibroblasts than in metformin treated fibroblasts. Furthermore, similarities were observed in chromosome territory positioning, with chromosome 10 moving to the periphery and 18 to the interior in response to either metformin or rapamycin treatment; however, chromosome 10 did not significantly change position in metformin treated cells indicating different effects of metformin vs. rapamycin.

**Figure 5:**
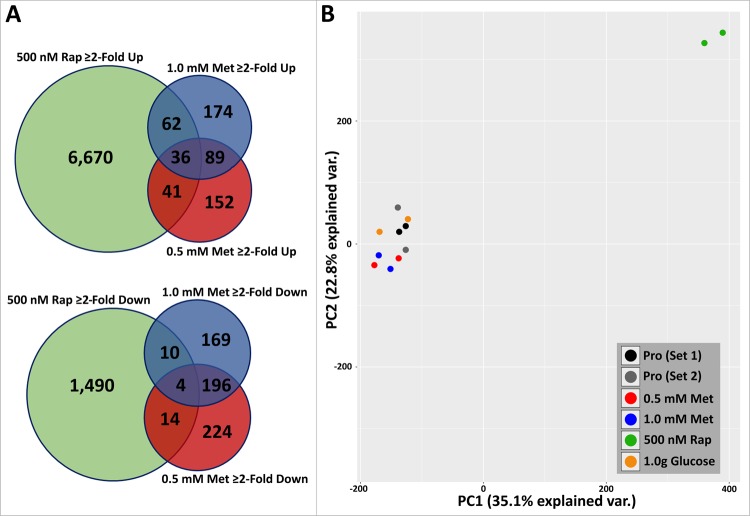
Genes changing ≥2-fold in response to 0.5 mM/1.0 mM metformin are divergent to those changing in response to 500 nM rapamycin treated fibroblasts. (**A**) Venn diagrams demonstrating the number of genes up-regulated (top) or down-regulated (bottom) between 0.5 mM (red), 1.0 mM (blue) metformin and 500 nM rapamycin (green). Numbers in each segment represent genes that are not shared. Genes in overlapping segments represent genes shared either between samples. (**B**) Principal Component Analysis (PCA) demonstrating the divergence between sample-sets. Two proliferative sets are included (Pro Set 1: Black; Pro Set 2: Grey) to represent controls in RNAseq assays for rapamycin-treated samples and for metformin or glucose deprived samples. Each circle represents an RNAseq replicate, with each sample having two identically coloured circles. A key is given in the bottom left corner corresponding circle colour to condition (0.5 mM Metformin (Met): Red; 1.0 mM Met (Blue); 500 nM Rap (Green); 1.0 g/L Glucose (Orange)). Y-axis represents PC2 (22% explained var.) and X-axis represents PC1 (35.1% explained var).

To determine if metformin and rapamycin have the same impact on gene expression and biological pathways in normal human cells, data from 2DD treated with 500 nM rapamycin were re-analysed with a ≥2-fold change in gene expression cut-off and compared to data from metformin-treated 2DD fibroblasts. Conditions and time points for these experiments were consistent except for drug treatment applied. Of the 6,809 genes up-regulated ≥2-fold by rapamycin, 62 overlapped with 0.5 mM metformin and 41 overlapped with 1.0 mM metformin treatments (Fig. 5A; Supplemental Table 5). Furthermore, only 36 up-regulated genes were common between all three conditions. Of the 1,518 genes ≥2-fold down-regulated in response to 500 nM rapamycin or 0.5/1.0 mM metformin treatment, we identified that 4 genes were common between all treatment conditions. Furthermore, only 10 genes down-regulated in 0.5 mM metformin-treated fibroblasts overlapped with genes down-regulated in response to rapamycin treatments. Similarly, only 14 genes down-regulated in response to 1.0 mM metformin were shared with those down-regulated in rapamycin-treated cells (Fig. 5A; Supplemental Tables 7, 8). Network analyses of these shared genes demonstrated poor connectivity, with subsequent pathway analyses demonstrated ≤3 nodes contributing to statistically significant pathways (p ≤ 0.05; FDR ≤ 0.05) (Supplemental Table 10). This comparison of transcriptome profiles indicates that metformin and rapamycin have significantly different impacts on genome function.

To further compare the response of metformin with rapamycin principal component analysis (PCA) was performed. In addition to the comparison of RNA-seq changes in gene expression between proliferative cells, 0.5 mM metformin, 1.0 mM metformin, 500 nM rapamycin treatments, we also included 2-fold changes in gene expression induced by reduced glucose to evaluate how closely related metformin and rapamycin treatments were to actual decreases in glucose in primary dermal fibroblasts. PCA analyses indicate that there are differences in gene expression profiles between proliferative, metformin treated (both at 0.5 mM and 1.0 mM) and glucose depletion, but 500 nM rapamycin induced a vastly different change compared to the others. (Fig. 5B). The closer relationship between glucose depletion and metformin indicates that metformin is having a similar impact on energy sensing and that rapamycin and metformin, although both considered to be mimetics of caloric restriction, have different impacts on gene expression profiles in primary fibroblasts.

### Changes in gene expression in response to metformin are regulated by the FOXO3a transcription factor

**Figure 6:**
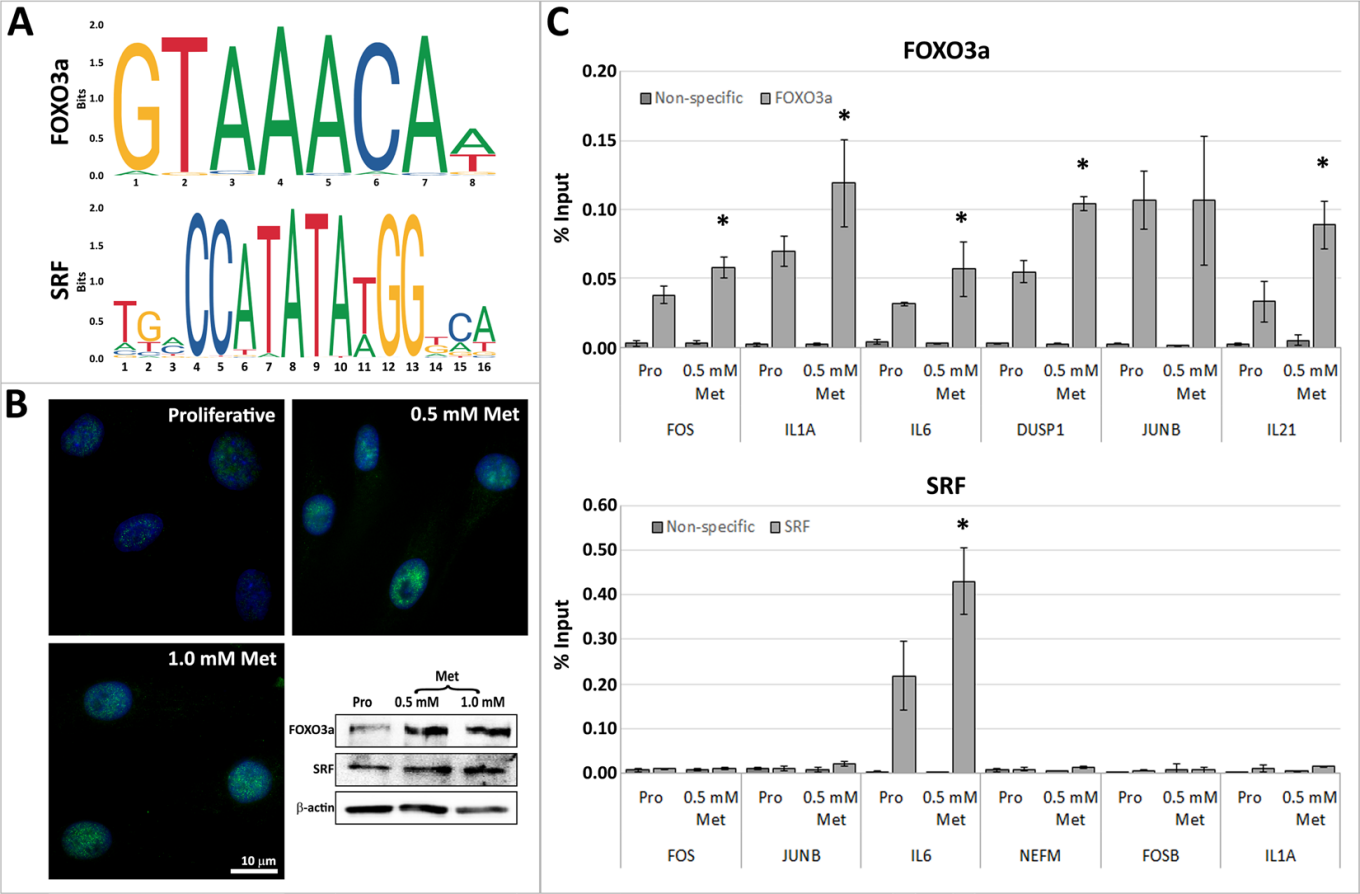
FOXO3a promoter occupancy is increased in genes up-regulated by 0.5 mM metformin treatments in primary human fibroblasts. (**A**) Using CLOVER, promoters of genes changing expression following metformin treatments had overrepresentation of FOXO3a and SRF transcription factor binding sites. Position weight matrices/sequence logos of these binding sites are shown. Log-base-2 of the information content of each nucleotide (Y-axis) and position of these nucleotides (X-axis) are given. (**B**) Immunofluorescence for FOXO3a (green) in proliferative, 0.5 mM and 1.0 mM metformin treated fibroblasts. Chromatin is counterstained with H33342 (blue). Scale bar = 10 μm. Western blot assays for FOXO3a (top), SRF (centre) and beta actin (bottom) are given for proliferative (pro) 0.5 mM and 1.0 mM metformin (met) treated whole protein lysates. (**C**) ChIP assays were used to compare promoter occupancy of FOXO3a (top) and SRF (bottom) in proliferative (dark grey) and 0.5 mM metformin treated (light grey) samples. Promoters of genes analysed are given (X-axis) and percent (%) enrichment over input reported (Y-axis). Error bars = S.E.M. *P < 0.05 by Students T-Test.

Previously we had identified that STAT5A/B was one of the major transcription factors driving changes in gene expression in response to rapamycin^20^. To identify the mechanism of metformin-mediated changes in gene expression, transcription factor motif searches using the *cis*-element over representation (CLOVER) algorithm^24^ were conducted on genes changing expression ≥2-fold. Analyses were conducted for a region spanning 1050 bp upstream of the transcription start site, revealing a number of overrepresented transcription factor binding sites (TFBS). In genes up-regulated ≥2-fold in response to 0.5 mM metformin, 226 over and under-represented binding motifs were identified (P < 0.00001), whilst in genes ≥2-fold down-regulated in response to 0.5 mM metformin, 433 over and under-represented binding motifs were proposed (P < 0.00001). In response to 1.0 mM metformin treatments, in genes ≥2-fold up-regulated, 110 potential TFBS were identified, whilst in genes ≥2-fold down-regulated, 202 potential TFBS were determined (p < 0.0001). Of the over-represented transcription factors in 0.5 and 1.0 mM metformin, FOXO3a had one of the highest raw scores and lowest p-values across up-regulated genes, indicating confidence in the presence of this promoter site by CLOVER analysis. Furthermore, FOXO3a has previously been implicated in modulating health and lifespan across species^26,28,29^, which is particularly interesting considering that metformin is a proposed anti-aging compound^12,13^. Several members of the FOXO transcription factor family were identified across samples and the presence of FOXO3 in 2DD fibroblasts confirmed through the high number of specific reads in the RNAseq datasets. Of the 361 up-regulated genes identified, 131 (36%) contained FOXO3a motifs (Fig. 6A), significantly above that expected by chance (p < 0.00001) in 0.5 mM metformin-treated fibroblasts. A second transcription factor, serum response factor (SRF), that has also been shown to decrease during aging^36^, was identified in response to metformin in up and down-regulated genes. Therefore, subsequent analyses were focused on FOXO3a and SRF in response to metformin.

SRF and FOXO3 exhibited no significant changes in gene expression (data not shown) by RNAseq. However, western blot analyses of FOXO3a and SRF in response to 0.5 mM and 1.0 mM metformin revealed increases in the levels of their expressed proteins. Additionally, immunofluorescence assays demonstrated increased number and size of foci of FOXO3a within the nucleus in response to 0.5 mM and 1.0 mM metformin treatments (Fig. 6B). To confirm the binding of FOXO3a to the promoters of genes up-regulated by 0.5 mM metformin, chromatin immunoprecipitation (ChIP) assays were conducted, with 5 of the 6 genes tested exhibiting significant increased binding of the FOXO3a transcription factors. SRF transcription factor binding was also examined; however, only 1 of the 6 genes tested (*IL-6*) demonstrated significant enrichment over input. Intergenic region of chromosome 18 was analysed as a binding control and demonstrated no enrichment. These results implicate FOXO3a in regulating cellular response to metformin treatments and because of its role in the regulation in cytokine expression they also suggest that cytokines may be important in fibroblast response to dietary restriction mimetics.

## Discussion

We have identified both changes in genome organization and function in response to metformin treatment in primary human fibroblasts. Initial data revealed that treatment of normal human foreskin fibroblasts with either 0.5 mM or 1.0 mM metformin in culture increased population doublings without evidence of cell death and indicate that treatments are functioning through the expected pathways, with increased AMPK activation. We further identified re-location of chromosomes 18 and 10 within the nuclear volume, with 1.0 mM metformin treatments inducing re-location of chromosome 18 only. Previously, we observed that rapamycin, a proposed mimetic of CR, also increased population doubling times; however, these data reveal that this increase is far more prominent than that observed with metformin at 120 h; changes in positioning of chromosomes 10 and 18 were similarly different, although importantly not as drastic^24^. Comparative analyses of RNAseq data from 120 h 500 nM rapamycin and metformin-treated 2DD revealed that the number of genes changing expression ≥2-fold in response to 0.5 mM (740) and 1.0 mM metformin (754) were far fewer than and had little overlap with those changing expression in response to 500 nM rapamycin (8327). Although both metformin and rapamycin reduced growth rates of proliferative cells, rapamycin had a much more dramatic impact, additionally inducing more substantial repositioning of chromosome territories when compared to metformin treated-fibroblasts. Gene expression profiles imply different mechanisms by which these changes are induced. How these cellular responses to both of these compounds translate to the tissue and organismal levels to result in increased health lifespan needs to be further investigated.

Transcriptome analyses revealed differing pathway enrichment in networks resulting from 0.5 mM and 1.0 mM metformin treatments. No pathways were detected in 1.0 mM metformin at FDR ≤ 0.05 whilst genes increasing ≥2-fold in 0.5 mM metformin treatments were enriched in numerous pathways, including the activator protein-1 (AP-1) transcription factor pathway and cytokine-cytokine receptor interaction. Rapamycin induced enrichment of the cytokine-cytokine receptor interaction pathway in up-regulated genes. Metformin treatment also induced up-regulation of cytokine genes and enrichment of the cytokine-cytokine receptor interaction pathway; however, the genes up-regulated were different and fewer than those up-regulated with rapamycin treatment. CLOVER analysis and ChIP assays identified FOXO3a as responsible for mediating metformin induced changes in gene expression, whilst STAT5A/B, proposed to be responsible for up-regulation of cytokines in response to rapamycin^24^, was absent in analyses based on metformin up-regulated genes. Gene set enrichment analyses therefore indicate that cytokines are important for response to both metformin and rapamycin; however, the regulation of these responses are independent of one another. Subsequent promoter analyses of genes increasing and decreasing expression ≥2-fold in both 0.5 mM and 1.0 mM metformin treatments revealed overrepresentation of the AP TFBS (transcription factor binding sites). The AP-1 complex is comprised of subunits of Jun (c-Jun, Jun B, Jun D) and Fos (c-Fos, Fos B, Fra-1 and Fra-2) transcription factor family members and is activated in response to numerous stressors (reviewed in^37^). Genes changing expression ≥2 fold by 0.5 mM metformin included members of this complex: *FOS, FOSB* (the proteins of which can dimerize with Jun proteins) and *JUNB*. AP-1 is known to regulate the expression of numerous genes, including cytokines^38,39^, and mediate cell proliferation and apoptosis. Decreases in AP-1 abundance and activity with age have been reported across species, including mouse lymphocytes^40^ and human diploid fibroblasts^41^. An increase in the AP-1 transcription factor pathway has not previously been reported in response to metformin in a primary cell line model; however, intermittent fasting in *C. elegans* revealed up-regulation of AP-1, with deletion resulting in a moderately decreased lifespan^42^. This could implicate AP-1 as an important transcription factor in regulating extension in health and lifespan in response to CR and CR mimetics, including metformin.

**Figure 7:**
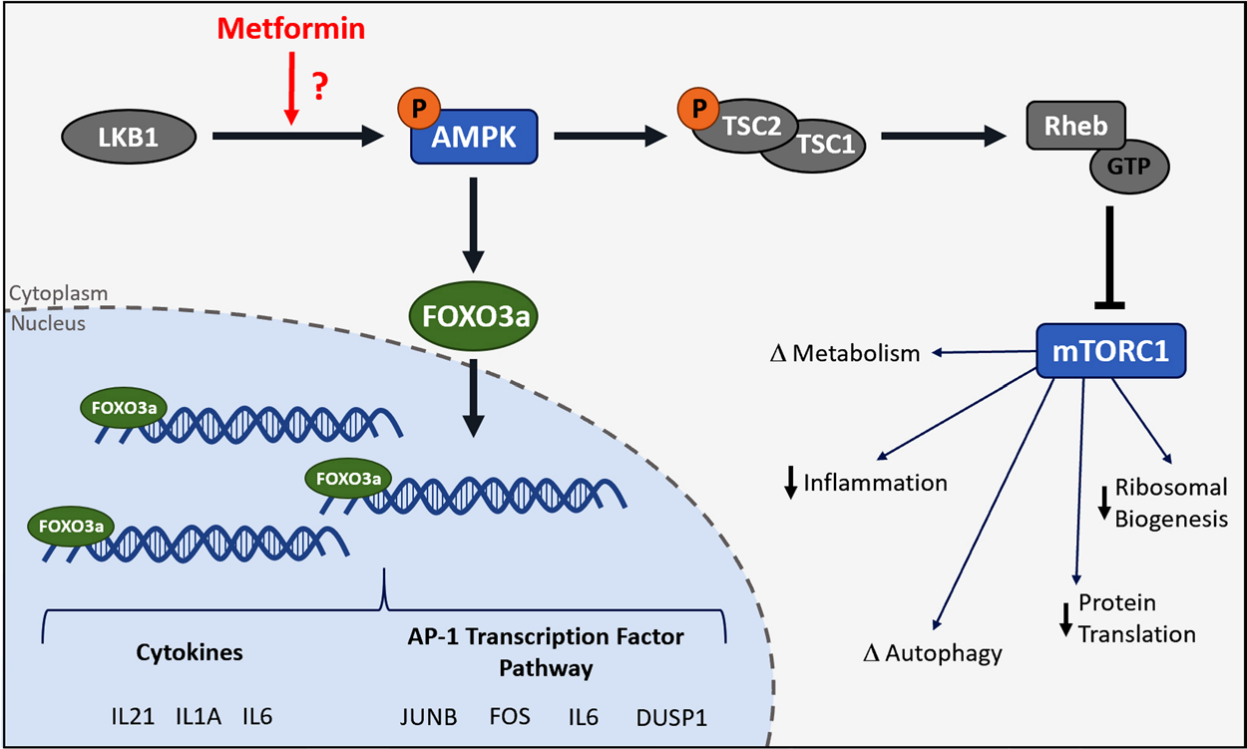
Proposed model for the impact of Metformin on FOXO3a and gene transcription in primary human fibroblasts. A model of the proposed impact of metformin on FOXO3a. In response to metformin treatment, it is likely that AMPK is phosphorylated and FOXO3a is translocated into the nucleus from the cytoplasm, resulting in increased transcription of cytokine genes and gens in the activator protein-1 transcription factor pathway. Simultaneously, as a result of AMPK activation, mTORC1 likely becomes inhibited, resulting in previously described changes in autophagy, protein translation, metabolism and other down-stream pathways.

FOXO TFBS were also overrepresented in response to metformin-treatment, with binding to the promoter sites of up-regulated genes confirmed by ChIP. FOXO proteins are evolutionarily conserved and play important roles in many cellular processes, including: cell cycle progression, metabolism control, stress responses and differentiation (reviewed in^29^). Furthermore, FOXO3a has been extensively linked to regulation of health and lifespan^29^. Although FOXO3a has been examined in its response to metformin numerous times in cancer-based cell lines^43–46^ and model organisms^47^, there appear to be no studies reporting the impact of metformin on FOXO3a in primary human cell-lines. FOXO3a is a known regulator of cytokine expression, with previous data indicating a role in the repression of the inflammatory response decreasing chronic inflammation; however, our data indicate up-regulation of cytokine genes with both pro- and anti-inflammatory activities (*IL-6, IL1-b, TNF, IL-21*) in response to metformin treatments. Cytokines are usually associated with inflammation and aging; however, many cytokines appear to have dual roles in maintaining cellular homeostasis. For example, even though IL-6 is frequently referred to as pro-inflammatory, it stimulates production of the IL-1 receptor antagonist (an anti-inflammatory cytokine)^48^. Furthermore, mice with IL-6 knock outs demonstrate decreased insulin sensitivity, glucose intolerance^49^ and late onset obesity^50^; whilst it has also been shown as critical in regeneration and protection of some tissues (*e.g*. intestinal epithelial cells)^51,52^. Therefore, pro-inflammatory cytokines up-regulated in response to metformin may be involved in a more complicated role of maintaining homeostasis of pro- and anti-inflammatory cytokines (see Fig. 7 a schematic representation of FOXO3a function). Furthermore, recent work has established that rapamycin treatment, depletion of specific nutrients or exercise (all mimetics of or forms of CR) can up-regulate gene expression of cytokine and inflammatory genes in primary^24^ and immortalized^53^ cell lines and in human athletes^54^.

Jun-N-terminal kinase (JNK) has previously been demonstrated to activate AP-1 and FOXO3a in response to intermittent fasting in *C.elegans*^42^ and *D. Melanogaster* retina^55^. It has been proposed that JNK signalling interacts with insulin/IGF signalling pathways to regulate FOXO in response to environmental stresses (reviewed in^56^). FOXO3a can also be phosphorylated and its activity enhanced by AMPK^57^, a protein that is activated by a change in AMP:ATP via metformin^17,58^. This upstream regulation of FOXO3a can promote its nuclear localization and transcriptional activity^59,60^. SRF was also overrepresented in our datasets; however, only 1 of 6 genes changing expression in response to metformin that we tested demonstrated increased SRF binding in the promoter region indicating that either this factor is already highly bound and that metformin did not increase its occupancy or that SRF is not regulated by metformin. SRF has also been shown to decrease with age, and knockout outs in mice demonstrate a premature aging phenotype^36^. SRF binds at serum response elements (SRE) in *JunB and c-FOS*. When examining human variants of FOXO3a, Flachsbart and colleagues observed that in FOXO3 associated with long-lived humans, a single nucleotide variant resulted in formation of an SRF binding site, resulting in competition for SRF binding between FOXO3a and IGF-1, with tissue specific disruption of IGF-1 resulting in decreased IGF-1 serum levels^61^. Given that increased IGF-1 levels normally decrease FOXO expression, this redistribution of SRF in a manner that reduces IGF-1 levels could be part of a complex mechanism involved in regulating cellular response to metformin. No changes in IGF-1 transcript levels were observed in these data in response to metformin; however, this does not rule out potential changes in protein translation. Furthermore, if IGF-1 protein levels are changing, potentially altering gene expression profiles, these changes are still part of a complex mechanism and down-stream changes responding to metformin-treatment.

In summary, we have demonstrated that metformin induced significant changes in the transcript profiles of normal human fibroblasts. Furthermore, although much work has been done on the mechanistic (directly/indirectly via the mTOR pathway) and synergistic effects of metformin and rapamycin, we have compared these treatments in an identical cell line to establish how well these compounds mimic one another. We have established that even though there were some commonalities between metformin and rapamycin treatments, transcript profiles were distinct from each other. Furthermore, the mechanisms driving these changes in gene expression were also different, potentially explaining synergistic effects between the two CR mimetics. We observed enrichment in AP-1 transcription factor and cytokine/chemokine expression and demonstrated increased FOXO3a promoter occupancy at these genes, identifying FOXO3a as a novel regulator of gene expression in response to metformin in primary human fibroblasts; however, it will be necessary to delineate the exact pathways by which metformin promotes and regulates these changes, with the complete mechanistic function of metformin on the human body and in treating T2D being a challenge for researchers for many decades^62,63^. As metformin is a proposed anti-aging compound and CR-mimetic, these findings have potential applications in influencing health and longevity and understanding the aging process in response to different pathway interactions beyond a cell-culture model.

## Supplementary information

Supplemental Information
