# Supplementary material for: Metformin induces the AP-1 transcription factor network in normal dermal fibroblasts
Source: Sci Rep. 2019 Mar 29;9:5369. doi: 10.1038/s41598-019-41839-1 (PMC6441003; doi:10.1038/s41598-019-41839-1)
Supplement: Supplementary file 1 — Supplemental Information [file 41598_2019_41839_MOESM1_ESM.pdf]

Metformin induces the AP-1 transcription factor network in normal dermal fibroblasts

Zoe E. Gillespie[1,2], Chenxuan Wang[1], Flaviu Vadan[3], Topaza Y. Yu[1], Juan Ausió[4], Anthony Kusalik[3], *Christopher H. Eskiw[1,2]

[1] Department of Food and Bioproduct Sciences, University of Saskatchewan, Saskatoon, Canada

[2] Department of Biochemistry, University of Saskatchewan, Saskatoon, Canada

[3] Department of Computer Science, University of Saskatchewan, Saskatoon, Canada

[4] Department of Biochemistry and Microbiology, University of Victoria, Victoria, Canada

***Corresponding Author**
Christopher H. Eskiw
c.eskiw@usask.ca
Department of Food and Bioproduct Sciences, University of Saskatchewan, Saskatoon, Canada
Room 6E08
52 Campus Drive
University of Saskatchewan
Saskatoon, SK, S7N 5A8
306-966-2454

Supplemental Information

Gene/Direction	Sequence (5’-3’)	Gene/Direction	Sequence (5’-3’)
**ACTC1 F**	TGATGAAGCCCAGAGCAAGA	**LIF R**	ACGATGGGGAGGAGAGACAA
**ACTC1 R**	TCTCCATGTCGTCCCAGTTG	**MAN1A1 F**	TCCACTGGAGCATTGGATCTT
**ANKRD1 F**	CCGATGGATCTGGTGCTACA	**MAN1A1 R**	CAGTGAAGGGAATGGAGCAGA
**ANKRD1 R**	TGCCTTCAAAATGCCAGTGA	**NEFM F**	GGTGGAGCGCAAAGACTACC
**CISH F**	ACTCTGCCCTGGCTTCTCTG	**NEFM R**	AGCGGCATTTGAACCACTCT
**CISH R**	CATCCAGGATTGCCTTGGTT	**NFATC2 F**	CTAGCCCAGCAGAGGCCTTA
**CRIM1 F**	TTCGGGATTTACGGAACCTG	**NFATC2 R**	CCAGCATGTGTGAGCTTGGT
**CRIM1 R**	CGCTTCGTACTCGGTGAGG	**NQO1 F**	CCCGAATTCAAATCCTGGAA
**DDIT4 F**	GGTGGAGGTGGGGGAATAGT	**NQO1 R**	GAGGCTGCTTGGAGCAAAAT
**DDIT4 R**	GGCCCCTAAGCCTTTGTTTC	**NR4A2 F**	AGCACAGGCTACGACGTCAA
**EFEMP2 F**	GCCCAAACCTGTGTCAACTTC	**NR4A2 R**	AGGTGGCTGTGTTGCTGGTA
**EFEMP2 R**	CGGTTCTCAGAGACCTGGATG	**PPARa F**	TGCCATTCAAGGAGATGTGG
**FAU F**	CGCATGCTTGGAGGTAAAGTC	**PPARa R**	CATCGAACTTGCCAGTCAGC
**FAU R**	TTCTCCTGTTTGGCCACCTTA	**PRDX5 F**	GCATAGTGAAGGCCCTGAATG
**FKBP10 F**	GCCGTGCTAATCTTCAACGTC	**PRDX5 R**	GGGGTGGAGGAAGTAATCTGG
**FKBP10 R**	GGTGGTCTCATTGCAGGTCTC	**PTGS2 F**	GCCTACTGGAAGCCAAGCAC
**FOS F**	CCCTCACCCTTTCGGAGTC	**PTGS2 R**	TAAAGGGACAGCCCTTCACG
**FOS R**	GGCCTCCTGTCATGGTCTTC	**RCAN2 F**	CACGTGTGCGACAGTGACAT
**HIF1a F**	TCACTTTGCCAGCTCAAAAGA	**RCAN2 R**	CAGGCAGCTCAGTTGGACAC
**HIF1a R**	ACCAACAGGGTAGGCAGAACA	**RGS4 F**	AGCAGGAAGACGCTCAGAGG
**HIST1H3A F**	TCCGCCGTTATCAGAAGTCC	**RGS4 R**	GGAATTCGCAAGCAGGAAAG
**HIST1H3A R**	GAGCTCTGGAAACGCAGGTC	**SAA1 F**	GAGTGGCAAAGACCCCAATC
**IL11 F**	CAGACTTCTGCCCTGGCTCT	**SAA1 R**	ACCCTCTCCCCGCTTTGTAT
**IL11 R**	CCCACCCCAACATGAAAAGT	**SPARC F**	TACATCGGGCCTTGCAAATAC
**IL8 F**	TGCAGTTTTGCCAAGGAGTG	**SPARC R**	GGTGACCAGGACGTTCTTGAG
**IL8 R**	TGATAAATTTGGGGTGGAAAGG	**TRXR1 F**	CTGCGTGTCCTGTGCTTACC
**LIF F**	AGATGTCTCCGGGCCCTTAT	**TRXR1 R**	TGCTGCCTGCCTTCTATTCA

Gene	Forward Primer (5'-3')	Reverse Primer (5'-3')	Amplicon (bp)
FOS	CTTAAGTCCTCGGGGTCCTG	GAGAACATTCGCACCTGGTT	157
DUSP1	GCTGTGCGAGCTGTATCACC	TCTTTGCTGTCCTCGACCAA	178
FOSB	TTCCCTCTGACGTCATTGCT	CGATCTGGTCACTTGCATGA	185
IL1A (1)	AAGGAACAATTGCAAAGCACA	GGTGGCCTTCACCTGAAAAT	210
IL1A (2)	GGACCAGAGAGAAGCCTGGT	ACCAGGCAACACCATTGAAG	190
IL21	GGGGCAGGGATTGATGGAGT	CAGGTAAGATGCCAGGGGAGG	195
IL6 (1)	AGTGGAGGGCTTGGAAAAAG	GAATGAACCAGGCCTCTGTG	232
IL6 (2)	TGCATGCCCTGATGTCCTAT	CGTGCACTGTGATCCGTCTA	203
JUNB (1)	CTCCCTGAAACCCCTCACTC	CTAGTCAGCCACGGAAGTGC	248
JUNB (2)	ACCCTCCCGATTTACAGTGC	CTGGAGTCCACTGGGACAAA	177
NEFM	CAAGCGCCTTACCTTCTGCC	GGAGATGAGCGCCGAGGAG	201

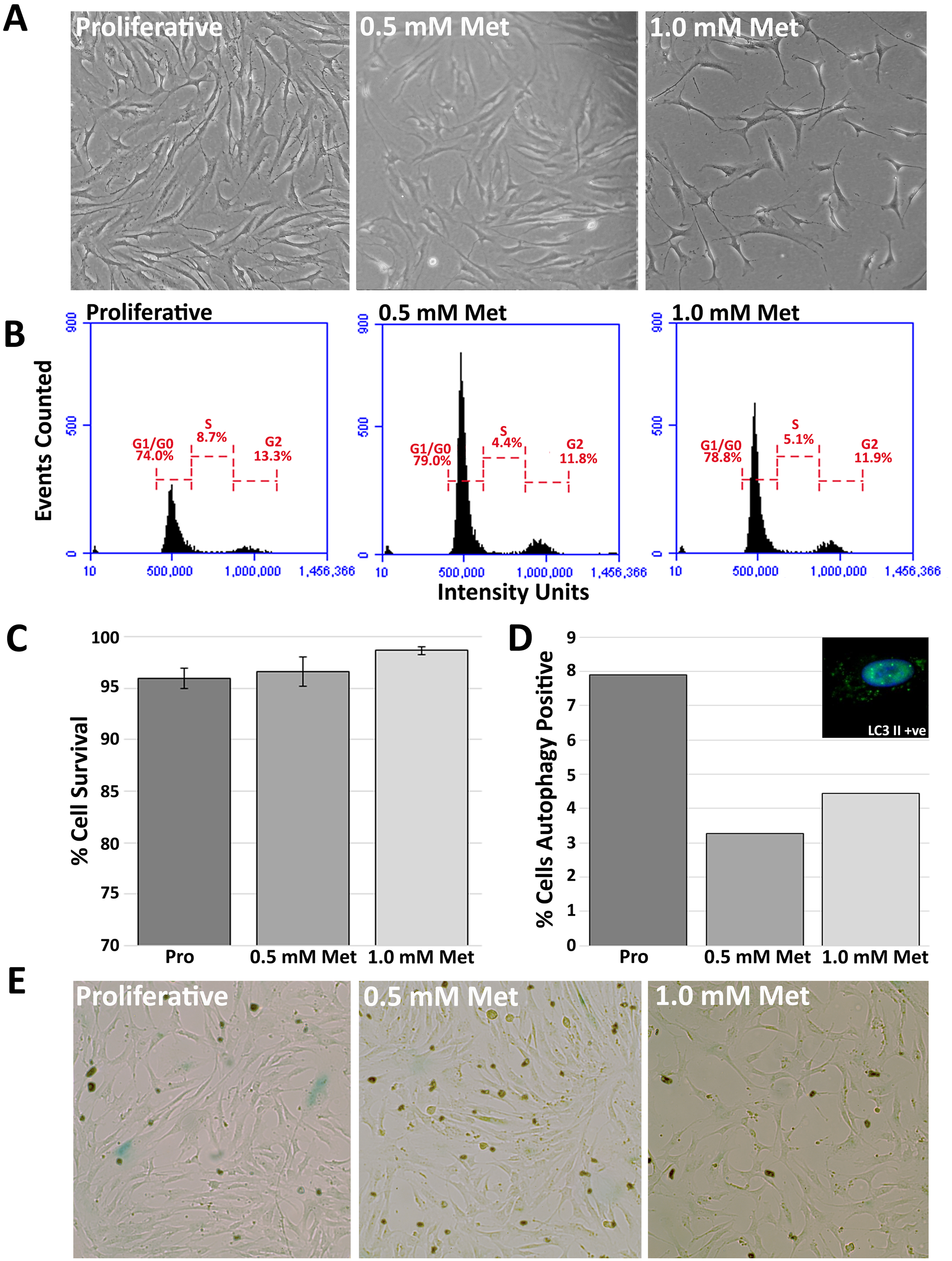

A
Proliferative
0.5 mM Met
1.0 mM Met
B
Proliferative
0.5 mM Met
1.0 mM Met
Events Counted
Intensity Units
G1/G0 74.0%
S 8.7%
G2 13.3%
G1/G0 79.0%
S 4.4%
G2 11.8%
G1/G0 78.8%
S 5.1%
G2 11.9%
0
500
900
10
500,000
1,000,000
1,456,366
C
% Cell Survival
100
95
90
85
80
75
70
Pro
0.5 mM Met
1.0 mM Met
D
% Cells Autophagy Positive
9
8
7
6
5
4
3
2
1
0
LC3 II +ve
Pro
0.5 mM Met
1.0 mM Met
E
Proliferative
0.5 mM Met
1.0 mM Met

0.5 mM Metformin ≥5-Fold Up-Regulated		0.5 mM Metformin ≥5-Fold Down-Regulated		1.0 mM Metformin ≥5-Fold Up-Regulated		1.0 mM Metformin ≥5-Fold Down-Regulated	
Gene Name	Fold Change	Gene Name	Fold Change	Gene Name	Fold Change	Gene Name	Fold Change
**FOSB**	13.99073	**PADI1**	-5.01038	**FOSB**	10.43015	**CRYM**	-5.27833
**FOS**	8.238771	**ANKRD1**	-5.09561	**RNVU1-7**	9.115089	**LRRC38**	-5.36819
**RP11-1100L3.8**	7.544827	**RP11-785D18.3**	-5.22942	**RNVU1-18**	6.920092	**RP11-588H23.3**	-5.39225
**KRT85**	6.126071	**HLA-U**	-5.83208	**RNU1-27P**	5.84307	**ANKRD1**	-5.41013
**NR4A1**	5.920966	**RP11-180C1.1**	-6.05292	**RNU1-28P**	5.544862	**CGB**	-5.68981
**ITGAL**	5.838044	**FABP3**	-8.67527	**RNU1-3**	5.415553	**RP11-180C1.1**	-5.76379
**ATF3**	5.752492	**LRRC38**	-12.9062	**FOS**	5.384613	**RP11-123O22.1**	-5.91358
**FOSB**	13.99073			**RNA5S15**	5.36764	**GS1-600G8.5**	-5.97031
**FOS**	8.238771			**ATF3**	5.139316	**AGT**	-6.04777
**RP11-1100L3.8**	7.544827			**RP11-1100L3.8**	5.061821	**RRAGD**	-6.20634
				**RNU1-1**	5.046011	**KCNK3**	-7.06061
						**FABP3**	-10.5548

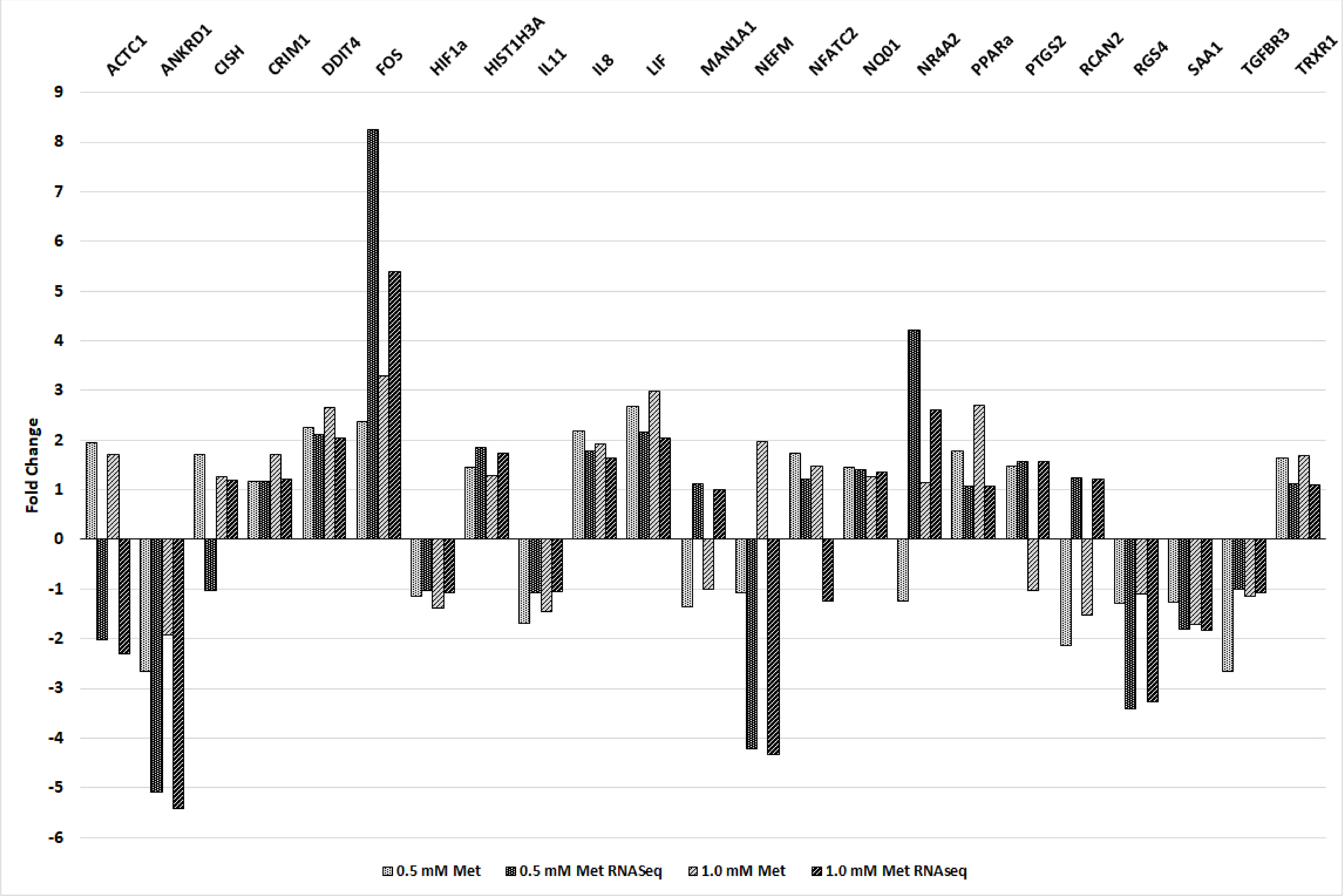
ACTC1
ANKRD1
CISH
CRIM1
DDIT4
FOS
HIF1a
HIST1H3A
IL11
IL8
LIF
MAN1A1
NEFM
NFATC2
NQ01
NR4A2
PPARa
PTGS2
RCAN2
RGS4
SAA1
TGFBR3
TRXR1
Fold Change
9
8
7
6
5
4
3
2
1
0
-1
-2
-3
-4
-5
-6
0.5 mM Met
0.5 mM Met RNASeq
1.0 mM Met
1.0 mM Met RNAseq

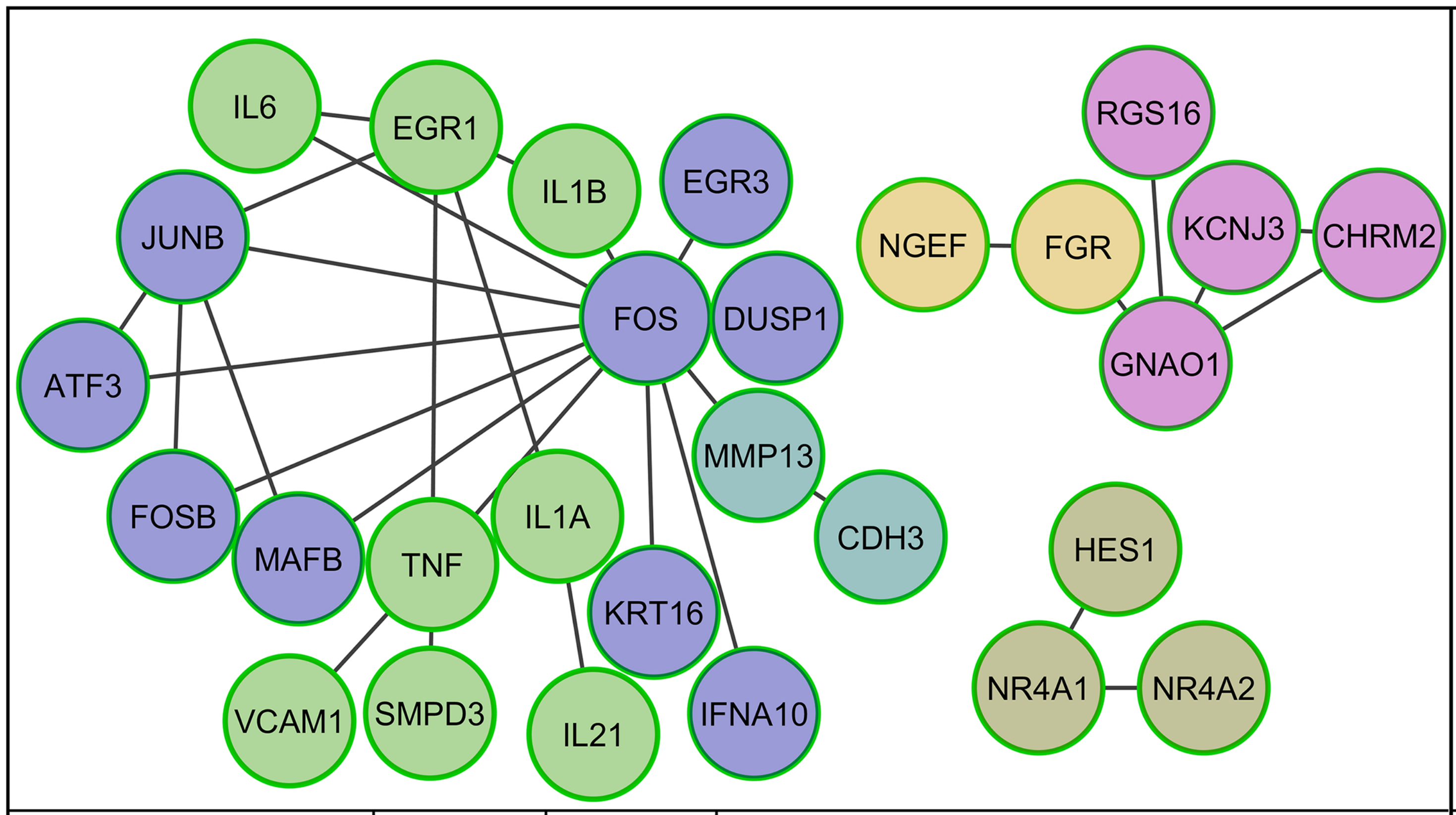

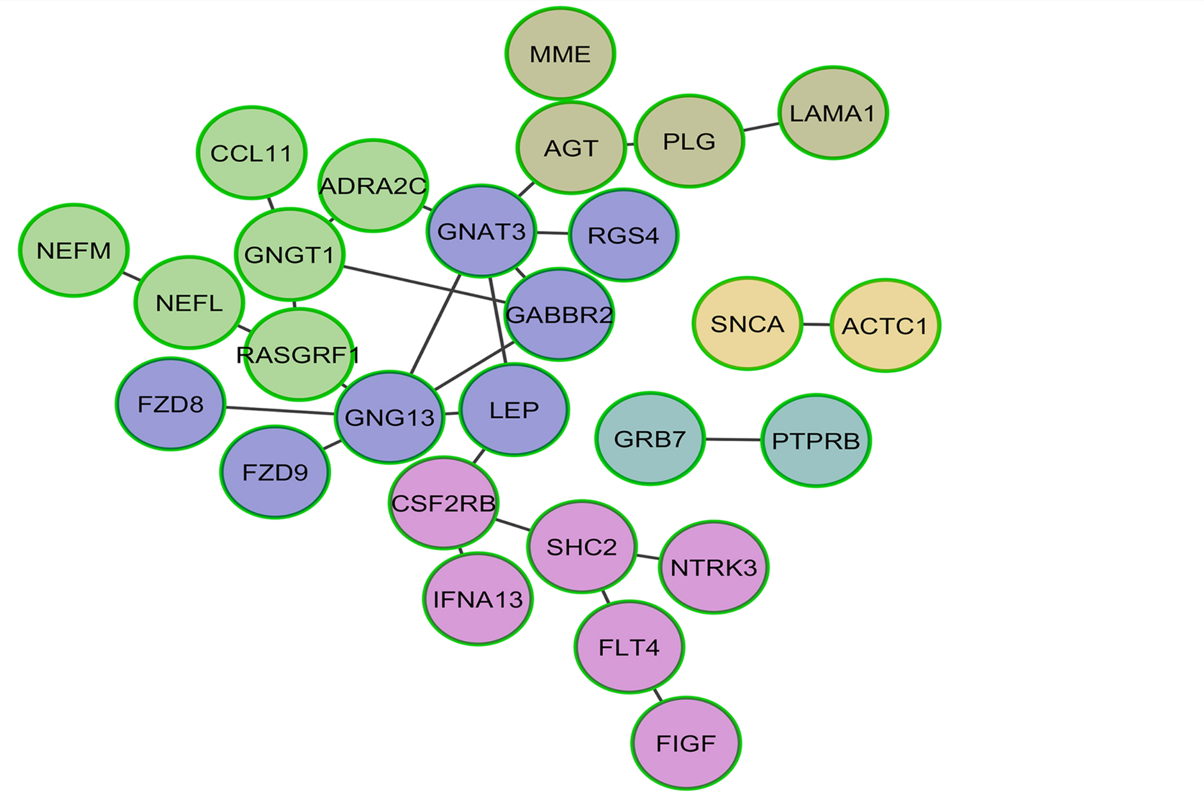

0.5 mM Met ≥2-Fold Up<br>Module Number	Nodes in Module	Node %	Node List
0	9	0.3214	ATF3, DUSP1, EGR3, FOS, FOSB, IFNA10, JUNB, KRT16, MAFB
1	8	0.2857	EGR1, IL1A, IL1B, IL21, IL6, SMPD3, TNF, VCAM1
2	4	0.1429	CHRM2, GNAO1, KCNJ3, RGS16
3	3	0.1071	HES1, NR4A1, NR4A2
4	2	0.0714	CDH3, MMP13
5	2	0.0714	FGR, NGEF

0.5 mM Met ≥2-Fold Down<br>Module Number	Nodes in Module	Node %	Node List
0	7	0.2593	FZD8, FZD9, GABBR2, GNAT3, GNG13, LEP, RGS4
1	6	0.2222	ADRA2C, CCL11, GNGT1, NEFL, NEFM, RASGRF1
2	6	0.2222	CSF2RB, FIGF, FLT4, IFNA13, NTRK3, SHC2
3	4	0.1481	AGT, LAMA1, MME, PLG
4	2	0.0741	GRB7, PTPRB
5	2	0.0741	ACTC1, SNCA

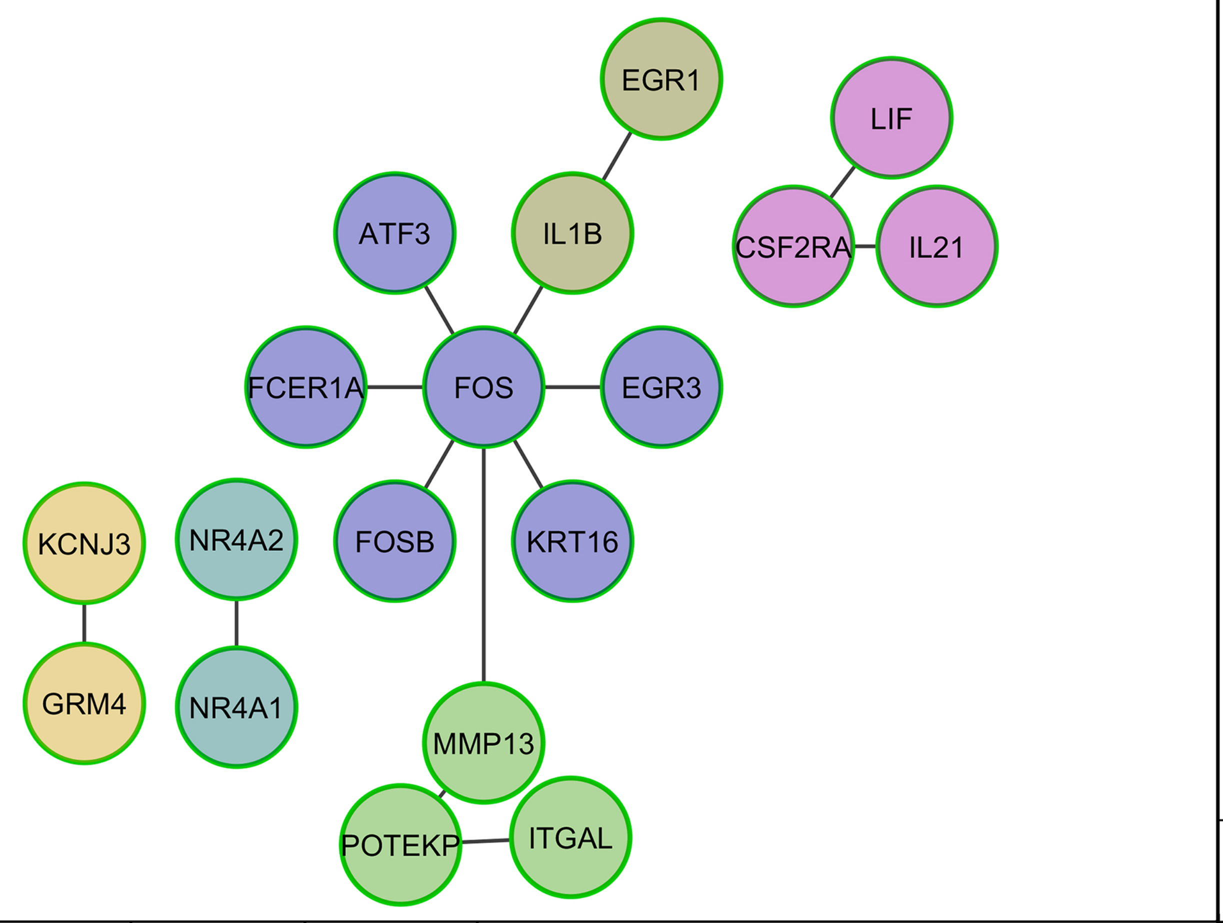

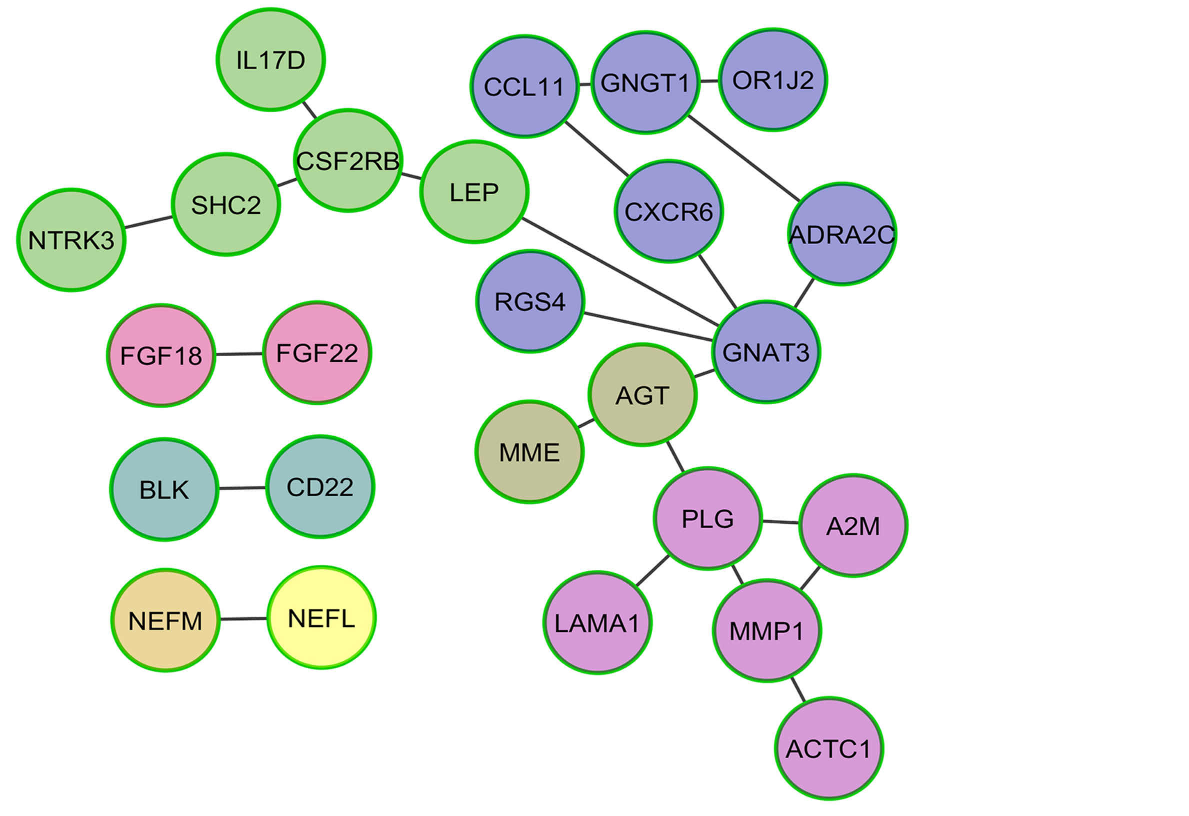

1.0 mM Met ≥2-Fold Up<br>Module	Nodes in Module	Node %	Node List
0	6	0.3333	ATF3, EGR3, FCER1A, FOS, FOSB, KRT16
1	3	0.1667	ITGAL, MMP13, POTEKP
2	3	0.1667	CSF2RA, IL21, LIF
3	2	0.1111	EGR1, IL1B
4	2	0.1111	NR4A1, NR4A2
5	2	0.1111	GRM4, KCNJ3

1.0 mM Met ≥2-Fold Down<br>Module	Nodes in Module	Node %	Node List
0	7	0.28	ADRA2C, CCL11, CXCR6, GNAT3, GNGT1, OR1J2, RGS4
1	5	0.2	CSF2RB, IL17D, LEP, NTRK3, SHC2
2	5	0.2	A2M, ACTC1, LAMA1, MMP1, PLG
3	2	0.08	AGT, MME
4	2	0.08	BLK, CD22
5	2	0.08	NEFL, NEFM
6	2	0.08	FGF18, FGF22

0.5 mM Met, 1.0 mM Met & 500 nM Rap (36 Genes)	0.5 mM Met & 1.0 mM Met (89 Genes)		0.5 mM Met & 500 nM Rap (62 Genes)	1.0 mM Met & 500 nM Rap (41 Genes)
NR4A2	TRGC2	ANKRD24	NTSR1	A4GNT
RP11-150C16.1	RP11-656A15.1	SH3TC1	FGR	RP11-62C3.8
CTD-2308B18.3	LINC00475	DHRS3	RP13-60M5.2	RP1-50J22.4
LINC01021	B3GALT5-AS1	RP11-563N4.1	GDF10	GPR62
NTRK2	LRCOL1	RAB39B	LONRF2	RP11-169F17.1
NR4A1	SLC9A2	ADM2	RP11-47J17.3	NONOP2
CTC-236F12.4	SEMA3B-AS1	FGF9	RP11-166B2.5	RP11-382E9.1
SBSPON	RP11-291L22.7	RP11-90K6.1	METTL24	AC009166.7
FOSB	KRT16	DLX3	HDAC11-AS1	HCG23
RP4-798A10.4	LRMP	LINC00989	SLC9C2	RP11-138B4.1
RGS16	RP11-585F1.6	RP11-463O9.9	MGAT3	AC008440.10
C8orf4	RP11-359D14.2	KRT85	IL21-AS1	ZNF793-AS1
EGR3	ADAMTS9-AS2	OR8S1	RP11-508N22.12	RP11-218E20.3
UPK1A-AS1	TMEM37	RP3-462E2.3	CYP21A2	CTD-2561J22.5
RP5-1103B4.3	ITGAL	NTSR1	RP11-849F2.9	GLP2R
KRTAP3-1	RP11-544M22.1	FGR	RIPPLY3	RP11-266L9.6
HEYL	SACS-AS1	RP13-60M5.2	ABCA8	RP11-24P14.1
UPK1A	RP11-430H10.4	GDF10	DUSP1	KRTAP5-AS1
BCL2L10	MMP13	LONRF2	IL6	C9orf106
NKX6-1	GYG2P1	RP11-47J17.3	LINC00519	RP11-290F24.3
LIF	DUOXA2	RP11-166B2.5	MUC19	RP1-102K2.8
BFSP2-AS1	KRT8P10	METTL24	LINC01059	SPNS3
SLCO6A1	DDX11L10	HDAC11-AS1	RP11-551L14.4	RP11-60A24.3
KCNJ3	LIPH	SLC9C2	RP3-465N24.6	CIB3
TCTE1	EGR1	MGAT3	RP11-20B24.7	EPHA5-AS1
IL1B	RP5-1021I20.1	IL21-AS1	RP11-624M8.1	U1
LINC00592	OR1J1	RP11-508N22.12	PRSS36	AF196972.9
TSPAN11	KB-1410C5.2	CYP21A2	AC016735.2	SLC8A3
AGTR1	KB-1836B5.1	RP11-849F2.9	IGF1	CTD-2185K10.1
RP11-173M1.4	H19	RIPPLY3	OXT	HMGA1P4
VCAM1	RP11-352D3.2	ABCA8	RP1-28O17.1	LRFN2
CCND2	AC138783.10	DUSP1	FENDRR	AZU1
CACNA1G-AS1	LINC01397	IL6	CTD-2537O9.1	RP5-899B16.1
RP11-1100L3.8	LINC01312	LINC00519	RPL9P28	AC009229.6
LHX9	LINC01503	MUC19	BFSP2	CTA-221G9.12
PAH	KRT18P38	LINC01059	GLDN	RP11-676J15.1
	DDIT4	RP11-551L14.4	IL1A	RP1-212P9.3
	FOS	RP3-465N24.6	AF196972.3	TAS1R1
	CCL8	RP11-20B24.7	AP001615.9	SRSF10P1
	FAM27C	RP11-624M8.1	SST	CSF2RA
	RP11-379K17.4	PRSS36	CTD-2302E22.4	RP11-700A24.1
	RP11-442H21.2	AC016735.2	RP1-292L20.3	
	AC007787.2	IGF1	KRTAP4-11	
	CYP2B6	OXT	RP11-136O12.2	
	SUSD2	RP1-28O17.1	TDRD6	
	GGT2	FENDRR	PCAT7	
	AC074011.2	CTD-2537O9.1	RP11-779O18.3	
	AADACL4	RPL9P28	RP11-61O11.1	
	ASTN2-AS1	BFSP2	MEOX1	
	RP11-932O9.8	GLDN	AC034243.1	
	CTB-174D11.2	IL1A	RP4-583P15.10	
	RP11-442N24__B.1	AF196972.3	SCRG1	
	RP1-199J3.5	AP001615.9	SGCG	
	RNA28S5	SST	AP004372.1	
	ENTPD2	CTD-2302E22.4	AC009120.10	
	CTD-2651B20.3	RP1-292L20.3	CTD-2377D24.8	
	RPL18AP7	KRTAP4-11	RP11-88E10.5	
	VN2R19P	RP11-136O12.2	CTD-2201G3.1	
	CTD-3032H12.2	TDRD6	CHRM2	
	RP11-71E19.2	PCAT7	C6orf25	
	CTD-2008E3.1	RP11-779O18.3	ST8SIA2	
	AC005076.5	RP11-61O11.1	SLC14A1	
	LL09NC01-139C3.1	MEOX1		
	IL21	AC034243.1		
	HAP1	RP4-583P15.10		
	ATF3	SCRG1		
	CYP21A1P	SGCG		
	RP11-63E9.1	AP004372.1		
	CCND2-AS1	AC009120.10		
	ZP1	CTD-2377D24.8		
	RP11-1105G2.4	RP11-88E10.5		
	RNF112	CTD-2201G3.1		
	MCHR1	CHRM2		
	CYYR1	C6orf25		
	ARMC12	ST8SIA2		

0.5 mM Met, 1.0 mM Met & 500 nM Rap (4 Genes)	0.5 mM Met & 1.0 mM Met (196 Genes)			0.5 mM Met & 500 nM Rap (10 Genes)	1.0 mM Met & 500 nM Rap (14 Genes)
RP11-981G7.1	CTD-2235C13.2	LINC01443	GRAMD4P8	SH2D2A	RP11-307C12.11
TRBV25-1	PADI1	CLDN14	C1orf110	ZNF962P	LINC00698
RP11-286H15.1	RP11-132A1.4	AQP7P3	NTRK3	RP11-552M11.8	PHACTR2P1
HRK	KCNK3	OPCML	RP11-814P5.1	RNF151	CYP4F25P
	AC079135.1	SRP68P2	AC005281.2	GRB7	RP11-755J8.1
	LEP	FZD8	TMEM158	RP11-386G11.3	SNX29P1
	IGHV3-49	IL1RN	ALG1L3P	RP11-368N21.5	RP11-229P13.25
	PPIAP13	RP11-785D18.3	SIRPAP1	DDX11L5	RP11-131L12.3
	SLC6A10P	PLG	PTGDS	GNG13	RP3-460G2.2
	RP13-631K18.2	RP11-501O2.5	LINC01087	RP11-22L13.1	AC019186.1
	EPHX3	RP11-672A2.4	RP4-734G22.3		RP11-769O8.2
	LINC01429	RP11-757G1.5	RP11-191N8.2		HMGN2P19
	PNMA6A	LRRN4CL	RP4-669L17.2		RP11-209M4.1
	DYSF	RPP25	RP11-180C1.1		Y_RNA
	RP1-239B22.5	RP11-38J22.1	DAZL		
	RP11-523H20.3	AL356585.2	RP11-59D5__B.2		
	MLLT4-AS1	SUN3	RP11-594N15.3		
	LINC01540	ANKRD20A8P	RP11-121C6.5		
	SLC6A15	CLEC12B	RP11-267N12.1		
	RP11-54A9.1	AP001189.4	PAPL		
	RP11-255M2.2	GOLGA8G	RNASE11		
	PRY	C15orf54	ASB18		
	RP11-123O22.1	ADAM11	PRYP3		
	RGS4	HSD17B14	PTAFR		
	SLC13A4	PPARGC1B	IL33		
	RP11-600K15.1	IVL	SLITRK1		
	ACTC1	RPLP0P2	HHIP		
	AGT	PVRL4	FABP4		
	RP11-400N13.3	RNU1-138P	RP11-960L18.1		
	AP006216.11	ALPL	GPRIN3		
	ASS1P5	NCR3LG1	CDCP1		
	BMP6	PNP	RRAGD		
	MAP3K7CL	MCTP1	RP11-355I22.5		
	AC008063.2	AGPAT9	TECRL		
	GOLGA8CP	ESM1	PRSS8		
	RP11-76K13.3	LINC01435	ANKRD1		
	PTGES2-AS1	ADRA2C	PAQR9		
	RP11-479J7.1	RENBP	TFPI2		
	TNFAIP8L3	S100P	C5orf52		
	GOLGA8EP	AC124914.3	AC003092.1		
	TACR2	GPNMB	SH2D5		
	SPP1	CLDN4	GALNT16		
	SLC25A34	RP11-148O21.3	HYALP1		
	RP11-392O17.1	CRYM	CSF2RB		
	NEFL	CTD-2066L21.1	GS1-600G8.5		
	RP11-497E19.2	RP11-203M5.8	RP11-390F4.6		
	CTC-512J12.7	AP004782.1	SH2D2A		
	CTSK	RP11-2K6.1	ZNF962P		
	IGFBP2	RTN4R	RP11-552M11.8		
	MSR1	LCE1B	RNF151		
	CALB2	XIRP1	GRB7		
	IGFN1	LRRC38	RP11-386G11.3		
	RP11-467L24.1	PRY2	RP11-368N21.5		
	PODXL	PRYP4	DDX11L5		
	RAPSN	ATP11A-AS1	GNG13		
	GS1-72M22.1	CD36	RP11-22L13.1		
	RP11-407P15.1	NEFM	RP11-14J7.6		
	LAMA1	AP000695.6	RP6-91H8.3		
	MME	PTPRB	GNAT3		
	FAM213A	RP11-108K3.1	AOC3		
	RP11-281A20.2	AC004744.3	FABP3		
	FAM230C	MME-AS1	RP11-297M9.2		
	BAIAP2L2	LINC00862	LINC01272		
	RP5-875H18.9	STON2	C10orf35		
	CTD-2218G20.2	RP11-588H23.3	SEMA7A		
	GNGT1	MYT1	RP11-281A20.1		
	ATP2A3	LRRC32	ACP5		
	PTGER4	C1QTNF9B	RP11-781P6.1		
	SHC2	CCL11			

Gene Set	Protein From Network	P-Value	FDR	Nodes
Pathway analysis of ≥2-fold up-regulated genes: shared between 0.5 mM and 1.0 mM metformin				
**AP-1 transcription factor network(N)**	4	8.58E-05	1.80E-02	ATF3, EGR1, FOS, FOSB
**HTLV-I infection(K)**	6	1.89E-04	1.80E-02	CCND2, ATF3, EGR1, VCAM1, ITGAL, FOS
**IL-17 signaling pathway(K)**	4	2.53E-04	1.80E-02	FOS, MMP13, IL1B, FOSB
**AGE-RAGE signaling pathway in diabetic complications(K)**	4	3.46E-04	1.80E-02	AGTR1, EGR1, VCAM1, IL1B
**Interleukin-4 and 13 signaling(R)**	4	4.76E-04	1.80E-02	VCAM1, LIF, FOS, IL1B
**TNF signaling pathway(K)**	4	4.76E-04	1.80E-02	VCAM1, LIF, FOS, IL1B
**Calcineurin-regulated NFAT-dependent transcription in lymphocytes(N)**	3	4.87E-04	1.80E-02	EGR1, EGR3, FOS
**Malaria(K)**	3	5.84E-04	1.87E-02	VCAM1, ITGAL, IL1B
**MAPK signaling pathway(K)**	5	1.46E-03	4.23E-02	FGF9, NR4A1, FOS, IL1B, NTRK2
Pathway analysis of ≥2-fold up-regulated genes: 0.5 mM metformin only				
**ATF-2 transcription factor network(N)**	4	2.14E-04	4.47E-02	JUNB, DUSP1, IL6, HES1
**Downstream signaling in naïve CD8+ T cells(N)**	4	3.10E-04	4.47E-02	JUNB, TNF, IFNA10, TNFRSF18
Pathway analysis of ≥2-fold up-regulated genes: 1.0 mM metformin only				
**Ribosome(K)**	16	1.11E-16	6.66E-15	RNA5S7, RNA5S8, RNA5S5, RNA5S6, RNA5S3, RNA5S4, RNA5S1, RNA5S2, RNA5S14, RNA5S15, RNA5S12, RNA5S13, RNA5S10, RNA5S11, RNA5S16, RNA5S17
**Ribosome biogenesis in eukaryotes(K)**	17	1.11E-16	6.66E-15	RMRP, RNA5S7, RNA5S8, RNA5S5, RNA5S6, RNA5S3, RNA5S4, RNA5S1, RNA5S2, RNA5S14, RNA5S15, RNA5S12, RNA5S13, RNA5S10, RNA5S11, RNA5S16, RNA5S17
**Ribosome(K)**	16	1.11E-16	6.66E-15	RNA5S7, RNA5S8, RNA5S5, RNA5S6, RNA5S3, RNA5S4, RNA5S1, RNA5S2, RNA5S14, RNA5S15, RNA5S12, RNA5S13, RNA5S10, RNA5S11, RNA5S16, RNA5S17

Gene Set	Protein From Network	P-Value	FDR	Nodes
**Aldosterone synthesis and secretion(K)**	3	1.68E-04	0.0111	NR4A2, NR4A1, AGTR1
**Cellular roles of Anthrax toxin(N)**	2	2.98E-04	0.0111	VCAM1, IL1B
**AGE-RAGE signaling pathway in diabetic complications(K)**	3	3.09E-04	0.0111	VCAM1, IL1B, AGTR1
**Interleukin-4 and 13 signaling(R)**	3	3.96E-04	0.0111	VCAM1, LIF, IL1B
**TNF signaling pathway(K)**	3	3.96E-04	0.0111	VCAM1, LIF, IL1B
**African trypanosomiasis(K)**	2	1.00E-03	0.024	VCAM1, IL1B
**Interleukin-10 signaling(R)**	2	1.79E-03	0.0349	LIF, IL1B

Supplementary Figure Legends

Supplemental Table 1: RT-qPCR Primers. Primer sequences used in the validation of 0.5 mM and 1.0 mM metformin RNA sequencing datasets. Forward (F) and reverse (R) primers are listed as 5'-3'.

Supplemental Table 2: ChIP Primers. Primers designed for use in Chromatin Immuno-Precipitation (ChIP) assays. Forward (F) and reverse (R) primers are given in 5'-3'. Amplicon size (bp) is listed.

Supplemental Figure 3: Cellular morphology and growth profiles of 2DD fibroblasts treated with metformin. (A) Representative, greyscale, light microscopy images for Proliferative, 0.5 mM metformin (Met)-treated and 1.0 mM Met-treated 2DD at 120 h. (B) Proliferative, 0.5 mM metformin (Met)-treated and 1.0 mM Met-treated 2DD at 120 h were fixed and stained with propidium iodide. DNA content was then measured by flow cytometry. Presented histograms shown events counted (Y-axis) and fluorescence signal intensity (Intensity Units, X-axis). Percentage of cells in G0/G1, S and G2 phase of the cell cycle have been gated (red bar) and identified for each condition. (C) Percent (%) cell survival using trypan blue of proliferative (Pro), 0.5 mM metformin (Met)-treated and 1.0 mM Met-treated 2DD at 120 h (X-axis). (D) Percent (%) Cells Autophagy Positive (Y-axis) determined by presence of autophagosomes identified using LC3I/II immunofluorescence for proliferative (Pro), 0.5 mM metformin (Met)-treated and 1.0 mM Met-treated 2DD at 120 h (X-axis). A representative autophagy positive nucleus (LC3 II +ve) is inset top right of the graph. Chromatin is counterstained with H33342 and autophagosomes (LC3 II) are in green. (E) Representative light microscopy images for β-galactosidase (stained Proliferative (3.48% β-gal positive), 0.5 mM metformin (Met) (3.02% β-gal positive) and 1.0 mM metformin (Met)-treated (3.85% β-galactosidase positive) fibroblasts at 120 h. Error bars = S.E.M. No significance by students T-test at $P < 0.05$.

Supplemental Table 4: Genes up or down-regulated ≥5-fold in 0.5 mM and 1.0 mM metformin treated 2DD fibroblasts.

Supplemental Figure 5: Validation of RNAseq datasets by RT-qPCR. RT-qPCR was conducted on a subset of 23 genes identified to have changes transcript profiles by RNAseq. This was conducted for both 0.5 mM (Lightly dotted bars) and 1.0 mM metformin-treated fibroblasts

(lightly dashed bars). The relative fold change (Y-axis) for each gene was calculated using the ΔΔCT method and five normalizing genes. RNAseq predicted fold changes are also listed for each condition (darker dotted and dashed bars). Gene names are listed at the top of the graph, whilst a key for 0.5 mM and 1.0 mM metformin values by qPCR and RNAseq is given at the bottom.

Supplemental Figure 6: Gene modules identified in genes changing expression ≥2-fold in response to either 0.5 mM or 1.0 mM metformin treatments. Top panels represent modules identified in genes changing expression ≥2-fold in response to 0.5 mM whilst bottom panels represent modules identified in genes changing expression ≥2-fold in response to 1.0 mM metformin. Panels to the left are modules from up-regulated genes whilst panels to the right are for down-regulated genes. Each circle is a node, with lines between nodes indicating relationships and colours indicating genes belonging to the same module. Tables in each panel identify the module, node in module, node % and give a node list, listing genes in the module.

Supplemental Table 7: Genes up-regulated ≥2-fold identified as overlapping between 0.5 mM metformin, 1.0 mM metformin and 500 nm rapamycin treated fibroblast datasets from RNAseq.

Supplemental Table 8: Genes down-regulated ≥2-fold identified as overlapping between 0.5 mM metformin, 1.0 mM metformin and 500 nm rapamycin treated fibroblast datasets from RNAseq.

Supplemental Table 9: Biological pathway enrichment of networks constructed from shared and not shared genes ≥2-fold upregulated or downregulated in response to 0.5 and 1.0 mM metformin. The enriched pathway annotation terms (GeneSet) are listed. P-value and false discovery rates are presented. Nodes lists genes from our data set present in the enriched pathway. Absence of pathways indicates no enrichment in a given dataset.

Supplemental Table 10: Biological pathway enrichment of ≥2-fold upregulated genes common to 0.5 mM metformin, 1.0 mM metformin and 500 nm rapamycin treated fibroblasts. No pathway enrichment was present in common ≥2-fold downregulated genes.
